# Supplementary material for: The bone ecosystem facilitates multiple myeloma relapse and the evolution of heterogeneous drug resistant disease
Source: Nat Commun. 2024 Mar 19;15:2458. doi: 10.1038/s41467-024-46594-0 (PMC10951361; doi:10.1038/s41467-024-46594-0)
Supplement: Supplementary file 1 — Supplementary Information [file 41467_2024_46594_MOESM1_ESM.pdf]

Supplementary: The bone ecosystem facilitates multiple myeloma relapse and the evolution of heterogeneous drug resistant disease.

Authors/affiliations

Ryan T. Bishop[1]*, Anna K. Miller[2]*, Matthew Froid[2,3], Niveditha Nerlakanti[1,3]Tao Li[1], Jeremy Frieling[1], Mostafa M. Nasr[1,3], Karl Nyman[1,3] Praneeth R Sudalagunta[4], Rafael Canevarolo[4], Ariosto Siqueira Silva[4], Kenneth H. Shain[1,5], Conor C. Lynch[1]+ and David Basanta[2]+

[1] Department of Tumor microenvironment and metastasis, H. Lee Moffitt Cancer Center and Research Institute, Tampa, Florida

[2] Department of Integrated Mathematical Oncology, H. Lee Moffitt Cancer Center and Research Institute, Tampa, Florida

[3] The Cancer Biology Ph.D. Program, University of South Florida, Tampa, Florida, USA.

[4] Department of Metabolism and Physiology, H. Lee Moffitt Cancer Center and Research Institute, Tampa, FL 33612, USA

[5] Department of Malignant Hematology, H. Lee Moffitt Cancer Center and Research Institute, Tampa, Florida

* These authors contributed equally as first authors

+ These authors contributed equally as senior authors.

Supplementary Methods

1. Hybrid Cellular Automaton Methods

We developed a hybrid cellular automaton (HCA) model to capture some of the key cellular and molecular populations that characterize the MM bone ecosystem. This model was developed from scratch although some of the assumptions, including those about what are the most important populations for the model, build on our previous experience building a similar model to study bone metastatic prostate cancer[1-3]. In this instance our code (available on this repository: https://github.com/dbasanta/MM_ABM) build on the HAL library which will help our code become a more self-sufficient and reusable platform of use to other bone-modelling scientists. We have generated different in vitro data and *in vivo* model to parameterize, calibrate the model and validate the results. Building on our experience starting computational models of cancer from homeostasis, this newer code is more robust and capable of performing bone remodeling simultaneously in several sites. This robustness is also demonstrated by the fact that homeostasis in the bone is preserved even with the use of variable depths of osteoclast-mediated bone resorption which was not the case previously.

The model we developed incorporates six different cell types, including precursor osteoclasts, active osteoclasts, mesenchymal stem cells (MSCs), precursor osteoblasts adult osteoblasts, and multiple myeloma (MM) cells. Additionally, we incorporate two signaling molecules including RANKL and bone derived factors (BDFs) such as transforming growth factor beta (TGF-β). In the sections below, we describe the specific assumptions and parameters incorporated into the HCA of normal bone remodeling (**Supplementary Section 1.1**), multiple myeloma (**Supplementary Section 1.2**), and bortezomib treatment (**Supplementary Section 1.3**). Refer to **Supplementary Tables 1-6** in **Supplementary Section 1.5** for a complete description of the parameters and values used in the HCA.

1.1. Normal Bone Remodeling

Algorithmic Implementation

Initial and boundary conditions of agent grid

We first develop a model of trabecular bone remodeling in which we represent a cross section of the bone marrow as a 2D rectangular grid. We define the length of the grid as $0 \leq x \leq 160\, px$ and the height as $0 \leq y \leq 150\, px$, where $\Delta x = 1\, px = 10\, \mu m$, the diameter of a myeloma cell. To obtain the initial arrangement of bone, we first initiated the bone as a $60\, px \times 50\, px$ rectangle in the center of the grid, surrounded by bone marrow. The initial area of the bone was set to represent the approximate bone area to total area (BA/TA) from sham limbs of wild type RAG2 mice (mean: 12.4%, n = 6, data not shown). As bone remodeling occurs over time, the initial rectangle is transformed, though the BA/TA remains approximately constant. We arbitrarily select one of these final bone configurations as the initial condition for subsequent runs of the model. Hence, trabecular bone initially consists of 12.9% of the total area. We assume that the number of precursor osteoclasts remains constant and is approximately equal to the number of monocytes, which is 2.8% of the bone marrow[4]. MSCs initially consist of 0.01% of the bone marrow[5]. We implement periodic boundary conditions on all sides of the domain for all cell types.

Timesteps

We use two separate time step sizes, Δt_{diff} and Δt_{cells}, to account for the differences in the timescales for diffusion (fast) versus cell processes (slow). We use $\Delta t_{diff} = 1\, sec$ and $\Delta t_{cells} = 6\, min$.

Simulation Process

The computational algorithm was implemented using the Hybrid Automata Library (HAL)[6] and is organized as shown in **Supplementary Fig. 1a** and **Fig. 1c**.

Precursor osteoclasts

Fusion

Precursor osteoclasts fuse and become multinucleated osteoclasts. This process is regulated by the expression of RANKL (**Supplementary Fig. 2f**), which is expressed by osteoblast lineage cells such as osteoblast precursors, osteocytes, and bone lining cells[7, 8]. It is not well understood which cell type is the primary source of RANKL for osteoclast fusion, but it has been suggested that microdamage to trabecular bone leads to osteocyte apoptosis, which may signal to bone lining cells to express RANKL[7, 8]. In our model, we assume that bone remodeling events are initiated through microfractures that are uniformly distributed over 4 years, the approximate turnover time of trabecular bone[9]. The total number of remodeling events was approximated using the initial total amount of bone divided by the approximate amount of bone resorbed per osteoclast. For simplicity, each basic multicellular unit (BMU) initiates from a single remodeling event that consists of five adjacent cells on the perimeter of bone that begin to express RANKL. The number of cells expressing RANKL was selected to equal the approximate diameter of an osteoclast, which is $50\ \mu m$[10].Precursor osteoclasts follow the gradient of RANKL and once there are five adjacent cells on the perimeter of bone, they fuse to become an active osteoclast (osteoclast) with probability

$$p_{fusion} = 1 - (1 - MAX\_FUSION\_RATE)^{TIMESTEP\_AGENT} \quad \#(1)$$

This probability reflects that fusion is a process that takes at least 3 days to complete. We note that this probability is scaled geometrically since the agent time step (6 min) is smaller than the unit of time (1 hour), hence the probability that the preosteoclast does not fuse per agent time step must be considered. Additionally, fusion requires stimulation with RANKL which is modeled as a Hill function such that the probability of fusion increases and saturates at one as RANKL increases (**Supplementary Fig. 2g**):

$$f_{fusion}(RANKL) = \frac{1}{1 + \left(\frac{K_{aOC}}{RANKL}\right)} \quad \#\ (2)$$

A Hill function is chosen as the functional form because it is commonly used to represent cell responses that are dependent on receptor-ligand interactions[11]. After the preosteoclasts fuse to become an osteoclast, five new preosteoclasts are randomly placed on the grid so that the number of preosteoclasts remains constant over time. To assess whether these functional forms qualitatively capture the dynamics of fusion in response to different concentrations of RANKL, we inhibit RANKL production by multiplying the production rate by scale factor π_{Ri} (**Equation 10**). As expected, the total number of osteoclasts decreases as RANKL decreases (**Supplementary Fig. 2h**).

Movement

Precursor osteoclasts migrate in response to a gradient of RANKL[12, 13]. We use the same technique to describe the probability of a cell remaining stationary or moving up, down, left, or right used in the original implementation of the hybrid discrete-continuum model[14] (**Equation 9**). If a preosteoclast would otherwise move in a direction that is occupied by a myeloma cell or precursor osteoblast, the two cells swap positions on the grid. This requirement underlies the assumption that fusion is not limited by spatial restrictions and is necessary in order to capture *in vivo* data showing that osteoclasts increase over time due to multiple myeloma.

Active osteoclasts

In the model, active osteoclasts consist of 5 subunits to represent the size difference between osteoclasts and other cell types and to account for the fact that they are the result of the fusion of 5 preosteoclasts. In order for the osteoclast to function as a single unit, once a subunit of an osteoclast is selected for a given timestep, all of the other subunits are collected and the action for each is defined to be the same for a given osteoclast.

osteoclast lifespan

We assume that the amount of bone that osteoclasts resorb is proportional to their lifespan, which we define to be normally distributed with a mean lifespan of 14 days[9], bounded between 7 and 21 days:

$$N(\mu_{OC}, \sigma_{OC}^2)\ within\ the\ interval\ (OC_{min}, OC_{max})\ \#(3)$$

To verify the implementation of this assumption in the model, we show that the average osteoclast lifespan per year remains constant over time (**Supplementary Fig. 1e**). The end of the bone resorption phase is followed by a reversal period, which couples bone resorption and bone formation. When an osteoclast dies, it is removed from the grid, and the cleared space is designated as eroded bone. Bone derived factors continue to be produced at a lower level for three days, which allows precursor osteoblasts to move and attach to the eroded surface. This release of BDFs could either be due to matrix metalloproteinase activity[15] or small osteoclasts that remain on the surface[16], though the source is not modeled explicitly.

Osteoclast resorption

We assume that each osteoclast resorbs bone in the direction with the maximum amount of bone. Osteoclasts resorb bone tissue in areas where there is a high degree of mechanical stress or strain. There are several proposed mechanisms governing how osteoclasts detect areas of bone resorption. Integrins may play a role in guiding the direction of bone resorption by regulating the formation and orientation of the sealing zone. Specifically, integrins on the leading edge of the osteoclast are thought to sense the mechanical properties of the bone tissue and orient the osteoclast towards areas of higher mechanical stress or strain[17]. This results in the formation of a sealing zone that is oriented in the direction of the mechanical stress or strain, allowing the osteoclast to efficiently remove bone tissue from areas that are under the greatest mechanical load. Another hypothesis is that osteoclasts may be guided by the orientation of collagen fibers in bone[18]. Collagen is the main structural protein in bone, and it forms a network of fibers that give bone its strength and resilience. Studies have suggested that osteoclasts may be able to sense the orientation of collagen fibers in bone through a range of receptors

and signaling pathways, and that this information may guide their resorption activity. Another hypothesis is that osteoclasts may be guided by chemical gradients of signaling molecules that are produced by other cells in the bone microenvironment[17]. By following these chemical gradients, osteoclasts may be able to target areas of bone where remodeling is needed.

To determine the direction, we check the Von Neumann neighborhood around each subunit of the osteoclast and record the total amount of bone in each direction. If more than one direction contains the maximum amount of bone, one of these directions is randomly selected. The bone is removed from the grid as it is resorbed and is replaced by the osteoclast. As bone is resorbed, bone derived factors are released from the bone matrix[16], which is modeled as a production term in **Equation 10**. Because the amount of bone that osteoclasts resorb is proportional to their lifespan, we verify that the average amount of bone resorbed per osteoclast per year remains constant over time (**Supplementary Fig. 1g**).

Mesenchymal stem cells (MSCs)

Recruitment

MSCs have been identified in various niches within the bone marrow, and their precise location and distribution are still under investigation. Some of the most common locations where MSCs are found in the bone marrow are the endosteal region, the perivascular region, and the central marrow region[19, 20]) MSCs are located in close proximity to the endosteal surface, where bone remodeling takes place, and the majority of MSCs are in contact with blood vessels or surrounded by pericytes and other undefined cells, which suggests that these cells are located in the perivascular niche[19, 21]. After an osteoclast fuses, an MSC is placed on the grid within a certain radius of the osteoclast (MSC_radius), provided there is not already an MSC within the neighborhood. This requirement ensures that MSCs are located adjacent to sites of bone remodeling to couple bone resorption with bone formation[22]. However, to preserve that approximately 0.01% of the bone marrow consists of MSCs[5], MSCs that are not within a certain radius of an osteoclast are

removed from the grid. Therefore, the number of MSCs fluctuates over time but is capable of returning to the initial condition.

Proliferation

We assume that MSCs divide only when BDF is above a certain threshold (BDF_{thresh}) (**Supplementary Fig. 2c**). The probability of cell division is

$$p_{div} = 1 - \left(1 - f_{div}(BDF)\right)^{TIMESTEP\_AGENT} \#\#(4)$$

where

$$f_{div}(BDF) = \begin{cases} 0 & if\ BDF < BDF_{thresh} \\ \dfrac{\rho_{MSC}}{1 + \left(\dfrac{K_{MSC}}{BDF}\right)^2} & if\ BDF \geq BDF_{thresh} \end{cases} \#4$$

When an MSC divides we assume it undergoes asymmetric division, producing a precursor osteoblast. The daughter cell is randomly placed in an empty grid cell within the Moore neighborhood of the MSC.

Movement

MSCs migrate in the direction of higher TGF-β when there is a cytokine gradient, otherwise they move randomly[16]. As before, we use the hybrid discrete-continuum technique to describe the probability of cell movement (**Equation 9**).

Preosteoblasts

Death

We assume that preosteoblasts die if they are not adjacent to eroded bone with probability

$$p_{death} = 1 - (1 - pOB\_DEATH)^{TIMESTEP\_AGENT} \quad \#(5)$$

or with probability one if $pOB_{age} \geq 42\ days$. Once a preosteoblast dies it is immediately removed from the grid.

Proliferation

We assume that high levels of BDF promote proliferation of preosteoblasts whereas low levels promote differentiation [23]. The probability of cell division is the same as it is for MSCs (**Equation 4**; **Supplementary Fig. 2c**), except that a preosteoblast divides symmetrically to produce another preosteoblast. As before, the daughter cell is randomly placed in an empty grid cell within the Moore neighborhood of the preosteoblast. To assess whether these model assumptions qualitatively capture the dynamics of proliferation in response to different concentrations of BDF, we inhibit BDF production by multiplying the production rate by scale factor π_{Ti} (**Equation 10**). As expected, the average proportion of preosteoblast over time decreases as BDF decreases (**Supplementary Fig. 2e**).

Differentiation

After the bone resorption phase, preosteoblasts attach to the eroded surface and differentiate due to differentiation signals provided by the exposed bone matrix[22]. In the model, preosteoblasts that are adjacent to eroded bone and exposed to low levels of bone derived factors for two weeks will differentiate into active osteoblasts (osteoblasts).

Movement

We assume the precursor osteoblasts that are not attached to eroded bone, move in response to a gradient of bone derived factors, which is modeled in the same way as for MSCs with the hybrid discrete-continuum technique (**Equation 9**).

Osteoblasts

Death

Active osteoblasts ultimately either undergo apoptosis, become osteocytes, or become quiescent bone-lining cells[9]. In the model, we assume that osteoblast death is proportional to the amount of bone that was resorbed by the osteoclast subunit that had occupied the space prior to the osteoblast (osteoclast_depth). Therefore, osteoblast

death is also proportional to the average time it takes for an osteoblast to form one unit of bone (basal_time). This assumption permits the coupling of bone resorption with bone formation, since the lifespan of an osteoblast controls how much bone the osteoblast forms. Thus, bone homeostasis is maintained even when the amount of bone resorbed by an osteoclast varies. Under normal conditions the lifespan of an osteoblast is approximately 3 months[9], which we reproduce in our model (**Supplementary Fig. 1f**). This equation describes how to define the lifespan of an aOB so that it lives long enough to replace the bone resorbed by an aOC (during homeostasis).

$$aOB\_DEATH = basal\_time \cdot aOC\_depth \# (6)$$

Units for equation 6:

- aOB_Death: days
- basal_time: days per unit of bone
- aOC_depth: units of bone

Alternatively, an active osteoblast may unintentionally get buried in bone by itself or other osteoblasts during bone formation. This is comparable to an osteoblast becoming an osteocyte, one of its potential cell fates. In the model, this occurs if the Von Neumann neighborhood of an osteoblast only contains bone or other osteoblasts. When an osteoblast is buried, this has possible consequences for bone homeostasis since the osteoblast did not form as much bone as it would have had it completed its full lifespan. To maintain a steady state of bone, the lifespan of the nearest osteoblast is increased to allow it to build more bone to compensate for the amount lost due to the buried osteoblast. This is a simplifying assumption of the model since biologically there are many factors involved to regulate the number and lifespan of osteoblasts. However, several studies have demonstrated the ability of osteoblasts, osteocytes and other cells of the BMU to sense and communicate with one another through juxtacrine and paracrine signaling to maintain bone homeostasis, through regulate cell proliferation, lifespan and activity [24-27].

Bone Formation

We assume that the time it takes for an osteoblast to form a unit of bone is dependent on the local concentration of BDF. To determine the functional relationship between BDF and mineralization rate we use *in vitro* data from MC3T3-E1 cells cultured in osteoblastic media with various concentrations of TGF-β or the TGF-β inhibitor, 1D11 (**Supplementary Methods 2.4**). This data shows how the mineralization rate compares to the control, specifically that it increases as TGF-β decreases (**Supplementary Fig. 2a**). We approximate this trend using an exponential decay function and extract the parameters using nonlinear least squares regression (**Supplementary Fig. 2b**):

$$f_{BF}(BDF) = (f_0 - Plateau) \cdot e^{-\delta_{BF} \cdot BDF} + Plateau \quad \#(7)$$

This equation is unitless since it represents fold change to bone mineralization time.

To determine the time that it takes an osteoblast to form one unit of bone as a function of BDF, we scale the time it takes an osteoblast to form one unit of bone in basal levels of BDF (basal_time) by **Equation 7**:

$$Mineralization_{Time(BDF)} = \frac{basal_{time}}{f_{BF}(BDF)} \quad \#(8)$$

Units for equation 8:

- Mineralization_Time: days per unit of bone
- basal_time: days per unit of bone

This scaling allows for mineralization to increase when BDF is below the basal level. In the model, the mineralization time for each osteoblast is updated after sufficient time has passed to form a unit of bone. To determine which direction to form bone, we impose a set of rules to try to prevent the osteoblast from becoming buried and to prevent the creation of large gaps in the placement of the bone. To do this, we first check the Von Neumann neighborhood of an osteoblast for empty space. If more than one empty space exists, the osteoblast will preferentially move to the location where it will remain in contact with at least two units of bone and is not adjacent to another osteoblast; if this is not possible, one of the empty spaces will be randomly selected. To form bone, the osteoblast moves to the empty grid cell and its previous location is replaced with a new unit of bone. Because the amount of bone that osteoblasts produce is proportional to their lifespan, we

verify that the average amount of bone produced per osteoblast per year remains constant over time (**Supplementary Fig. 1h**). To assess whether these model assumptions qualitatively capture the dynamics of mineralization in response to different concentrations of BDF, we inhibit BDF production by multiplying the production rate by scale factor π_{Ti} (**Equation 10**). As expected, BDF has a biphasic effect on bone in which low BDF results in bone loss due to lack of MSC/preosteoblast proliferation and medium BDF results in bone growth due to increased mineralization (**Supplementary Fig. 2d**).

Movement

In the model, MSCs, preosteoblasts, and MM cells migrate in the direction of higher TGF-β when there is a cytokine gradient, otherwise they move randomly[22]. preosteoclasts migrate in response to a gradient of RANKL[12, 13]. We use the technique for motility described in the original implementation of the hybrid discrete-continuum model[14] to describe the probability of each cell being stationary (P_0), moving left (P_1), right (P_2), down (P_3), or up (P_4):

$$\begin{aligned} P_0 = 1 - \frac{4kD_n}{h^2} - \frac{k\chi}{h^2}\left(m_{i+1,j} + m_{i-1,j} + m_{i,j+1} + m_{i,j-1} - 4m_{i,j}\right) P_1 \\ = \frac{kD_n}{h^2} - \frac{k\chi}{4h^2}\left(m_{i+1,j} - m_{i-1,j}\right) P_2 = \frac{kD_n}{h^2} + \frac{k\chi}{4h^2}\left(m_{i+1,j} - m_{i-1,j}\right) P_3 \\ = \frac{kD_n}{h^2} - \frac{k\chi}{4h^2}\left(m_{i,j+1} - m_{i,j-1}\right) P_4 \\ = \underbrace{\frac{kD_n}{h^2}}_{Diffusion\,Term} + \underbrace{\frac{k\chi}{4h^2}\left(m_{i,j+1} - m_{i,j-1}\right)}_{Chemotaxis\,Term} \quad \#(9) \end{aligned}$$

where the subscripts specify the location on the grid, i.e., $x = ih$, $y = jh$ where $\{i, j\}$ are positive integers, k denotes the timestep, $k = \Delta t$, and h denotes the spatial step, $h = \Delta x = \Delta y$. In the absence of a gradient of chemokine m, where $m = \{R_L, T_\beta\}$, cell movement is random with diffusion coefficient D_n, whereas in the presence of a gradient cell movement is directed with chemotaxis coefficient χ.

Cytokines (RANKL and BDF)

Receptor activator of NF-κB ligand (RANKL; R_L) is a cytokine that is produced in both soluble and membrane-bound forms and can activate osteoclasts. Recently, osteocytes have been identified as a major source of RANKL[28, 29]. Once a microfracture occurs, RANKL is produced at the rate α_R at five adjacent cells on the perimeter of bone, which then diffuses in two spatial dimensions and degrades at the rate δ_R.

Bone derived factors (BDFs; T_β) consist of the group of cytokines that are released during bone remodeling, including TGF-β and IGF. These cytokines are produced in a latent form by many other cell types, including T-cells, macrophages, and platelets[30]. Instead of modeling the production of BDF explicitly by these other cell types, we assume that BDFs are constantly produced at rate α_B, and further produced at rate α_T while bone is resorbed by osteoclasts. We assume that BDF also diffuses in two spatial dimensions and degrades at rate δ_T.

$$\frac{\partial R_L(x,y,t)}{\partial t} = \underbrace{D_R\left(\frac{\partial^2 R_L}{\partial x^2}+\frac{\partial^2 R_L}{\partial y^2}\right)}_{Diffusion} + \underbrace{\pi_{Ri}\alpha_R B_{i,j}}_{Production} - \underbrace{\delta_R R_L}_{Decay} \quad \frac{\partial T_\beta(x,y,t)}{\partial t} = \underbrace{D_T\left(\frac{\partial^2 T_\beta}{\partial x^2}+\frac{\partial^2 T_\beta}{\partial y^2}\right)}_{Diffusion} + \underbrace{\pi_{Ti}\alpha_B}_{Basal\ Production} + \underbrace{\pi_{Ti}\alpha_T B_{i,j} C_{i,j}}_{Production} - \underbrace{\delta_T T_\beta}_{Decay} \quad \#(10)$$

where

$$\Omega_{i,j} = \{ 1 \quad if\ \Omega\ at\ (x_i, y_j) \ \ 0 \qquad otherwise \quad \Omega = bone\ cell\ (B)\ or\ osteoclast\ (C)$$

Boundary conditions of PDE grid

The system of PDEs is solved using the forward time centered space (FTCS) scheme with periodic boundary conditions imposed on all sides of the domain, which is implemented using the diffusion function in HAL. The timestep is chosen to ensure numerical stability, i.e., $\frac{D\Delta t_{diff}}{(\Delta x)^2} < \frac{1}{4}$.

1.2. Incorporating Multiple Myeloma

Preosteoclasts

Fusion

Multiple myeloma cells enhance RANKL expression in the bone microenvironment which leads to increased osteoclastogenesis[31]. In the model, we assume that if there are three or more myeloma cells within the Moore neighborhood of a bone cell located on the perimeter of bone, a new bone remodeling event is initiated. As described above, each bone remodeling event is characterized by RANKL expression from five adjacent cells on the perimeter of bone.

Mesenchymal stem cells (MSCs)

Recruitment

Multiple myeloma cells produce chemoattractants that recruit MSCs to the bone marrow[32]. We assume that one MSC is recruited per 50 myeloma cells, and that once it is recruited, an MSC remains in the bone marrow (data not shown).

Proliferation

Myeloma cells inhibit the differentiation of mesenchymal stromal cells through soluble factors and cell-cell contract[33]. In the model, we assume that the differentiation of MSCs to precursor osteoblasts occurs through asymmetric division. To capture the effect of myeloma cells on osteoblast differentiation, we assume that MSCs do not divide if they are within a certain radius of a myeloma cell (MM_radius).

Preosteoblasts

Differentiation

Myeloma cells inhibit the differentiation of osteoblast progenitors through soluble factors and cell-cell contract[22]. In the model, we assume that preosteoblasts do not differentiate if they are within a certain radius of a myeloma cell (MM_radius),

Multiple Myeloma

Initiation

A single myeloma cell is recruited within a certain radius of an osteoclast after the first osteoclast fusion event occurs.

Death

The bone marrow microenvironment supports the growth and survival of multiple myeloma cells[34]. To determine where myeloma cell death and division occur spatially in the microenvironment, we measured the distance of these cells to the nearest bone (**Supplementary Methods 2.8**; **Fig. 3a-d**). Because there is less cell death close to bone, we assume that multiple myeloma cell death (sensitive and resistant cells) is dependent on the local concentration of BDF. The probability of cell death is

$$p_{death} = 1 - (1 - MM_{DEATH})^{TIMESTEP_{AGENT}} \quad \#(11)$$

where

$$MM_{DEATH} = \{ \; MM\_DEATH \quad if\ BDF < BDF_{thresh} \; MM\_DEATH\_BDF \quad if\ BDF \geq BDF_{thresh} \; \#$$

Proliferation

We assume that myeloma cell division is dependent on the concentration of BDF and has maximum rate ρ_X. This maximum rate is dependent on if the myeloma cell is sensitive or resistant, since we showed that the U266 cell line has a cost of resistance (**Supplementary Fig. 7a-b**). Furthermore, we and others have shown that myeloma cell division increases near MSC/preosteoblast[35] (**Fig. 3f** and **Fig. 6a**). To incorporate this into the model, we assume that sensitive cells within a certain radius of an MSC or preosteoblast divide faster than sensitive cells that are not close to an MSC or

preosteoblast (**Fig. 3g**), which divide faster than resistant cells. We define the probability of cell division with the following Hill function:

$$f_{div}(BDF) = \frac{\rho_X}{1 + \left(\frac{K_X}{BDF}\right)^2} \quad \#(12)$$

where $\rho_X = \{MAX\_MM\_DIV, MAX\_MM\_DIV\_MSC, MAX\_R\_MM\_DIV\}$ and $K_X = \{K_{MM}, K_{MM\_MSC}, K_{R\_MM}\}$ depending on if the myeloma cell is sensitive, resistant, and/or within a certain radius of an MSC or preosteoblast (protect_radius). A Hill function is chosen as the functional form because it is commonly used to represent cell responses that are dependent on receptor-ligand interactions[11].

1.3. Incorporating Bortezomib

Preosteoclasts

Fusion

Bortezomib inhibits osteoclast differentiation *in vitro* in a dose-dependent manner[25]. To incorporate this into the model we multiply the probability of fusion (**Equation 2**) by a repressive Hill function so that the probability decreases as dose increases (**Supplementary Fig. 3d**):

$$f_{fusion\_BTZ}(RANKL, Dose) = f_{fusion}(RANKL) \cdot \frac{1}{1 + \left(\frac{Dose}{K_{dose}}\right)^2} \quad \#(13)$$

As expected, the total number of osteoclasts decreases as dose increases (**Supplementary Fig. 3g**).

Osteoblasts

Bone Formation

Bortezomib enhances the mineralization rate of osteoblasts *in vitro* in a dose-dependent manner (**Supplementary Fig. 3b**). To incorporate this into the model we scale the mineralization time (**Equation 8**) linearly so that at maximum dose (Dose = 1) the time it takes an osteoblast to form a unit of bone is reduced by half (**Supplementary Fig. 3c**):

$$Mineralization\_Time\_BTZ(BDF, Dose) = \frac{Mineralization\_Time(BDF)}{Dose + 1} \#(14)$$

As expected, the BA/TA increases as dose increases (**Supplementary Fig. 3f**) due to increased production of bone per osteoblast.

Multiple Myeloma

<u>*Death*</u>

We assume that Bortezomib treatment does not affect the probability of cell death for resistant cells. On the other hand, Bortezomib decreases viability for sensitive cells *in vitro* and slows growth *in vivo* in a dose-dependent manner (**Supplementary Fig. 3a** and **Fig. 6c**)[36]. *In vivo*, failure to eradicate the disease may be due to both cell intrinsic mechanisms such as mutations, alterations in signaling pathways, copy-number alterations, epigenetic changes, and cell extrinsic mechanisms such as environment mediated drug resistance (EMDR)[37]. EMDR is when myeloma cells are transiently protected from Bortezomib due to factors or interactions with other cells in the bone microenvironment, which we define in our model to be when bone derived factors are above a certain threshold or MSCs or preosteoblasts are within a certain radius of a myeloma cell[32]. The probability of cell death during Bortezomib treatment is (**Supplementary Fig. 3i**):

$$p_{death} = 1 - (1 - MM_{DEATH})^{TIMESTEP\_AGENT} \#(15)$$

where

$$MM_{DEATH} = \{ \ MM\_DEATH \qquad if\ RESISTANT\ and\ BDF$$
$$< BDF_{thresh}\ \ MM\_DEATH\_BDF \qquad if\ RESISTANT\ and\ BDF$$
$$\geq BDF_{thresh}\ \ MM\_DEATH(1 + 1.5 \cdot Dose) \qquad if\ SENSITIVE\ and\ BDF$$
$$< BDF_{thresh}\ and\ no\ MSC$$
$$/pOB\ \ MM\_EMDR\_DEATH \qquad if\ SENSITIVE\ and\ BDF$$
$$\geq BDF_{thresh}\ or\ MSC/pOB\ \#$$

Therefore, tumor response to Bortezomib treatment depends on dose as well as the presence of bone derived factors or MSCs/preosteoblasts. At high dose, it is possible to completely eradicate the tumor in the absence of EMDR, similar to what is observed in *in vitro* experiments (**Supplementary Fig. 3j**).

Proliferation

Once treatment is initiated, myeloma cells have a probability of developing resistance (p_{Ω}) during cell division that would cause the cell to become resistant to bortezomib.

Dose selection

Bortezomib

We define dose to be between 0 and 1, where 0 represents no treatment and 1 represents the maximum dose, i.e., the dose that is sufficient to kill off sensitive cells without having a major impact on normal bone cells such as MSCs and preosteoblasts. Based on the *in vitro* cell viability assay, we define maximum dose to be 10 nM (**Supplementary Fig. 3a**).

1.4. Parameter Exploration

Parameter estimates used in the HCA model may vary widely based on the experimental design. For example, MM cell proliferation and death rates vary depending on the MM cell line used, growth media, and/or cell-cell interactions. To test whether our results are dependent on a particular parameter value, select parameters were varied to assess how the relapse time (when MM burden reached 20% of the marrow) and proportion of sensitive cells at endpoint changed under continuous bortezomib treatment

(**Supplementary Fig. 10**). We found that the parameter controlling the impact of EMDR had a more striking effect on the proportion of sensitive cells at endpoint instead of the relapse time (**Supplementary Fig. 10b**), whereas the parameter controlling the cost of resistance had an impact on both outputs in the presence of EMDR (**Supplementary Fig. 10c**). Furthermore, the parameter controlling the survival advantage of BDF under no BTZ treatment conditions had only a minor impact on either output (**Supplementary Fig. 10d**), whereas the parameter controlling the proliferative advantage of sensitive cells in the presence of MSC/preosteoblast had a strong effect on the proportion of sensitive cells at endpoint. However, when the proliferative advantage is sufficiently strong, minimal residual disease may be present in the presence or absence of EMDR, though the proportion is higher with EMDR (**Supplementary Fig. 10e**). This analysis shows that although the proportion of sensitive cells at endpoint varied, our finding that EMDR maintains a reservoir of sensitive cells was robust for these parameters. Furthermore, we showed that EMDR consistently led to a higher proportion of tumors that relapsed, except when the proliferative advantage due to MSC/preosteoblast was sufficiently large.

1.5. Parameters

The HCA model contains a large number of parameters as it has six different cell types and two microenvironmental factors. Many of the parameters were difficult to obtain and had to be estimated. Because many of the cell actions, such as proliferation and chemotaxis, result from the combination of parameters rather than a single value, our goal is to capture key outputs such as bone remodeling homeostasis instead of precisely parameterizing the model.

Supplementary Table 1: Grid Parameters

Grid Parameter	Description	Value	Units	Source
$\boldsymbol{SPACESTEP}$ $(\Delta \boldsymbol{x})$	Space step of HCA grid	10.0	μm	Estimated*
$\boldsymbol{TIMESTEP\_AGENT}$ $(\Delta \boldsymbol{t_{cells}})$	Cell timestep	0.1	$hour$	Assumed

$\boldsymbol{N\_TIMESTEP\_PDE}$	Number of diffusion timesteps per agent timestep	360	number	Assumed
$\boldsymbol{TURNOVER\_TIME}$	Turnover time of trabecular bone	35040	$hours$	[9]
$\boldsymbol{xDim}$	Length of HCA grid	1600	μm	Assumed
$\boldsymbol{yDim}$	Height of HCA grid	1500	μm	Assumed

- *SPACESTEP*. We assume that $1\ pixel\ =\ 10\ \mu m$, the diameter of a myeloma cell (experimentally derived)

Supplementary Table 2: BDF/RANKL Parameters

BDF/RANKL Parameter	Description	Value	Units	Source
---	TGF-β in bone	0.2	ng/mg	[38]
$\boldsymbol{TGFB\_DiffCoef}$ $(\boldsymbol{D_T})$	BDF diffusion coefficient	780	$\mu m^2/min$	[39-42]
$\boldsymbol{RANKL\_DiffCoef}$ $(\boldsymbol{D_R})$	RANKL diffusion coefficient	780	$\mu m^2/min$	Assumed
$\boldsymbol{TGFB\_productionRate}$ $(\boldsymbol{\alpha_T})$	BDF production rate	2.04×10^{-9}	$(ng/\mu m^3) \cdot cell_{unit}^{-1} \cdot min^{-1}$	Estimated*
$\boldsymbol{RANKL\_productionRate}$ $(\boldsymbol{\alpha_R})$	RANKL production rate	2.04×10^{-9}	$(ng/\mu m^3) \cdot cell_{unit}^{-1} \cdot min^{-1}$	Assumed $\alpha_R = \alpha_T$
$\boldsymbol{TGFB\_basalRate}$ $(\boldsymbol{\alpha_B})$	BDF basal production rate	$2.04\ \times 10^{-11}$	$(ng/\mu m^3) \cdot min^{-1}$	Estimated*
$\boldsymbol{TGFB\_decayRate}$ $(\boldsymbol{\delta_T})$	BDF decay rate	0.35	min^{-1}	[43]
$\boldsymbol{RANKL\_decayRate}$ $(\boldsymbol{\delta_R})$	RANKL decay rate	0.35	min^{-1}	Assumed $\delta_R = \delta_T$
$\boldsymbol{maxTGFB}$ $(\boldsymbol{BDF_{max}})$	Maximum BDF at given location	8.7×10^{-10}	$ng/\mu m^3$	Estimated
$\boldsymbol{Ts}$ $(\boldsymbol{BDF_{basal}})$	Basal BDF	$\dfrac{\alpha_B}{\lvert\delta_T\rvert \cdot BDF_{max}}$	$unitless$	Estimated
$\boldsymbol{TGFBthresh}$ $(\boldsymbol{BDF_{thresh}})$	Threshold BDF	$1.05 \cdot BDF_{basal}$	$unitless$	Assumed
$\boldsymbol{maxRANKL}$ $(\boldsymbol{RANKL_{max}})$	Maximum RANKL at given location	1.7×10^{-9}	$ng/\mu m^3$	Estimated

$\boldsymbol{Extra\_TGFBtime}$	Duration of BDF expression during reversal phase	72	$hours$	Assumed
$\boldsymbol{max\_RANKL\_on}$	Maximum duration of RANKL	336	$hours$	Assumed

- *TGF-β diffusion rate,* D_T. The $780\ \mu m^2/min$ was estimated using the molecular weight of TGF-β (25 kDa [39]). A molecule of molecular weight (M) 0.3-0.5 kDa has a diffusion coefficient of approximately $10^{-6}\ cm^2/s$ [40]. The plot of $log\ log\ (D)$ vs. $log\ log\ (M)$ correlates with a line of slope $-\frac{1}{3}$ [41]. Thus, we solve

  $(D)\ = (10^{-6})\ + \frac{1}{3}((0.5)\ - (25)\ )$

  to get $D = 2.6\ \times\ 10^{-7}\ cm^2/s\ =\ 1560\ \mu m^2/min$. Small molecules diffuse through the extracellular space with an effective diffusion coefficient that is two to three times less than the free diffusion coefficient[42], thus $D^* = D/2 = 780\ \mu m^2/min$.
- *TGF-β production rate,* α_B.
  - Volume of resorption pit: $\pi r^2 h\ =\ \pi(25^2)(10)\ =\ 19634.95\ \mu m^3/day$, where r = radius of osteoclast and h = amount of bone resorbed per day
  - Rate of bone resorption by a $50\ \mu m$ OC: Given the density of bone = $1500\ kg/m^3$ [44], $(1500\ kg/m^3)\ \times\ (19634.95\ \mu m^3/day)\ \times\ (10^{-18}\ m^3/\mu m^3)\ =\ 2.945\ \times\ 10^{-11}\ kg/day\ =\ 2.945\ \times 10^{-5}\ mg/day$
  - TGF-β released/day due to bone resorption by a 50 μm OC: Given concentration of TGF-β in bone is 0.5 ng/mg [38], $(2.945\ \times\ 10^{-5}\ mg/day)\ \times\ (0.5\ ng/mg)\ =\ 1.47\ \times 10^{-5}\ ng/day$. Because a $50\ \mu m$ OC takes up 5 pixels, we divide this by 5 to get: $2.94\ \times\ 10^{-6}\ ng \cdot cell_{unit}^{-1} \cdot day^{-1} =\ 2.04\ \times\ 10^{-9}\ ng \cdot cell_{unit}^{-1} \cdot min^{-1}$, consistent with [45]. We convert this to units of ng per volume to be consistent with the units of chemotaxis, which requires dividing the amount (ng) by the soluble volume of each grid point. Because the soluble volume fraction is unknown, we set $\alpha_T = 2.04\ \times\ 10^{-9}\ (ng/\mu m^3) \cdot cell_{unit}^{-1} \cdot min^{-1}$, which was calibrated to reproduce the steps of normal bone remodeling.

- *TGF-β basal production rate,* α_B. We assume that BDF is produced at a higher rate through bone resorption compared to other sources in HCA[22]
- *TGF-β decay rate,* δ_T*:* The half-life of *TGF-β* is 2 minutes[44]. Thus, the decay rate is $\frac{ln ln\ (2)}{2} = 0.35\ min^{-1}$.
- maxTGFβ was determined by running the unnormalized model a number of times and then recording the maximum TGFβ at a single grid point. This value of BDF was then set

as maxTGFβ. The maximum TGFβ is printed after each model is run (tmax) so that it can be monitored whether the value stays between 0 and 1.

- Ts is the basal BDF and is defined as written in the table, $\alpha_B/(|\delta_T| \cdot BDF_{max})$ .This is the amount of BDF that is always present on the grid.
- MaxRANKL was determined by running the unnormalized model a number of times and then looking at the output "preosteoblastborn.csv" to evaluate the maximum value of RANKL that occurred when an osteoclast formed. This value of RANKL was then set as maxRANKL so that most values of RANKL that lead to the formation of an osteoclast are between 0 and 1 (however, it is possible that some values in future runs of model are not strictly below 1).

Supplementary Table 3: preosteoclast/osteoclast Parameters

preosteoclast/ osteoclast Parameter	Description	Value	Units	Source
$\boldsymbol{pOC\_DiffCoef}$	preosteoclast random motility	0.3	$\mu m^2/min$	Estimated
$\boldsymbol{pOC\_TaxisCoef}$	preosteoclast chemotaxis coefficient	5×10^{10}	$\mu m^2 min^{-1}(ng/\mu m^3)^{-1}$	Estimated
$\boldsymbol{K_{aOC}}$	Half-maximal concentration constant	0.01	$concentration$	Assumed
$\boldsymbol{\mu_{OC}}$	Mean osteoclast lifespan	14	$days$	[9, 46]
$\boldsymbol{\sigma_{OC}}$	osteoclast lifespan standard deviation	7/3	$days$	Assumed
$\boldsymbol{OC_{min}}$	Minimum osteoclast lifespan	7	$days$	Assumed
$\boldsymbol{OC_{max}}$	Maximum osteoclast lifespan	21	$days$	Assumed
$\boldsymbol{MAX\_FUSION\_RATE}$	Maximum fusion rate	$1/72$	$hours^{-1}$	[47]
$\boldsymbol{Unit\_RESORPTION}$	Time to resorb one unit of bone	1	day	[48]
$\boldsymbol{aOC\ diameter}$	Diameter of osteoclast	50	μm	[10]

- *Random motility coefficient of cell, preosteoclast_DiffCoef*: Stokes-Einstein relation: $D = \frac{k_B T}{6\pi\eta a}$. Given $k_B = 1.38 \times 10^{-23}\ N \cdot m, T = 310\ K$ (body temp: $310\ K$; room temp: $300\ K$), $\eta = 10^{-3}\ N \cdot$

s/m^2 (viscosity of water), $a = 5\ \mu m$ (cell radius), $D = 4.54 \times 10^{-10} cm^2 s^{-1} = 3\ \mu m^2/min$. We assume that preosteoclast random motility is 10-fold slower in bone marrow compared to water.

Supplementary Table 4: MSC/preosteoblast/osteoblast Parameters

MSC/preosteoblast/osteoblast Parameter	Description	Value	Units	Source
$\boldsymbol{MSC\_DiffCoef} = \boldsymbol{pOB\_DiffCoef}$	MSC/preosteoblast random motility	0.03	$\mu m^2/min$	Estimated
$\boldsymbol{MSC\_TaxisCoef}$	MSC chemotaxis coefficient	5×10^9	$\mu m^2 min^{-1} (ng/\mu m^3)^{-1}$	Estimated
$\boldsymbol{pOB\_TaxisCoef}$	preosteoblast chemotaxis coefficient	5×10^{11}	$\mu m^2 min^{-1} (ng/\mu m^3)^{-1}$	Estimated
$\boldsymbol{K_{MSC}} = \boldsymbol{K_{pOB}}$	Half-maximal concentration constant	$\sqrt{3} \cdot BDF_{thresh}$	$unitless$	Assumed
$\boldsymbol{MAX\_MSC\_DIVISION\_RATE} = \boldsymbol{MAX\_pOB\_DIVISION\_RATE}$ $(\boldsymbol{\rho_{MSC}} = \boldsymbol{\rho_{pOB}})$	Maximum proliferation rate	$1/24$	$hour^{-1}$	Assumed
$\boldsymbol{pOB\_DEATH}$	preosteoblast death rate	$1/72$	$hour^{-1}$	Assumed*
$\boldsymbol{pOB\_DIFF}$	preosteoblast differentiation time	336	$hour$	Determined from experiments
$\boldsymbol{MSC\_radius}$	Radius around osteoclast/MM to recruit MSC	40	μm	Assumed*
$\boldsymbol{MM\_radius}$	Radius of MM effect on MSC/preoste	80	μm	Assumed*

	oblast differentiation			
$\boldsymbol{Y0}$ $(\boldsymbol{f_0})$	Maximum fold change	5.055	$unitless$	Estimated*
$\boldsymbol{Plateau}$	Minimum fold change	0.5379	$unitless$	Estimated*
$\boldsymbol{scalefactor}$	Scale factor	$\frac{19.2}{BDF_{basal}}$	$unitless$	Estimated*
$\boldsymbol{k}$ $(\boldsymbol{\delta_{BF}})$	Fold change decay constant	$0.1136 \cdot scalefactor$	$unitless$	Estimated*
$\boldsymbol{basal\_time}$	Basal unit bone formation time	9	$days$	[49]

- *Random motility coefficient of cell, MSC_DiffCoef, preosteoblast_DiffCoef*: We assume that MSC and preosteoblast random motility is 100-fold slower in bone marrow compared to water, and 10 fold slower than preosteoclast.
- *Chemotaxis coefficient, MSC_TaxisCoef, preosteoblast_TaxisCoef*: The chemotaxis coefficient of human neutrophils in response to the tripeptide FNLLP is $150\ cm^2 s^{-1} M^{-1} \approx 5.18 \times 10^{-2} mm^2 (pg/mm^3)^{-1} day^{-1} = 3.6\ \times 10^{13} \mu m^2 (ng/\mu m^3)^{-1} min^{-1}$ (using 25 kDa as the molecular weight of TGF-β)[50, 51]. However, to give reasonable model outputs required us to calibrate this parameter further and assume that MSC_TaxisCoef < preosteoblast_TaxisCoef.
- *preosteoblast death rate, preosteoblast_DEATH*: Assumed to be same as preosteoclast[47]
- *MSC recruitment radius, MSC_radius*: Defined so that MSC is close enough to respond to BDF generated by osteoclast
- *MM radius of impact on MSC/preosteoblast, MM_radius*: Defined so that MSC/preosteoblast responds to MM even when not directly adjacent. This assumption was necessary since HCA assumes MM is unable to migrate towards MSC/preosteoblast in absence of BDF, which created distance between MM and MSC/preosteoblast.
- *Exponential decay* (**Equation 7**)*: Y0, Plateau, k*: These values were estimated by using an exponential decay function and nonlinear least squares regression with *in vitro* data from MC3T3-E1 cells cultured in osteoblastic media with various concentrations of TGF-β or the TGF-β inhibitor, 1D11 (**Supplementary Methods 2.4**).

Supplementary Table 5: MM Parameters

MM Parameter	Description	Value	Units	Source
$\boldsymbol{MM\_DiffCoef}$	MM random motility	0	$\mu m^2/min$	Assumed
$\boldsymbol{MM\_TaxisCoef}$	MM chemotaxis coefficient	5×10^9	$\mu m^2 min^{-1} (ng/\mu m^3)^{-1}$	Estimated
$\boldsymbol{MM\_DEATH}$	MM basal death rate	$1/120$	$hour^{-1}$	Assumed*
$\boldsymbol{MM\_EMDR\_DEATH}$	MM death rate with EMDR	$1/120$	$hour^{-1}$	Assumed*
$\boldsymbol{MM\_DEATH\_BDF}$	MM death rate with BDF	$1/1200$	$hour^{-1}$	Assumed*
$\boldsymbol{MAX\_MM\_DIVISION\_RATE}$	Maximum proliferation rate	$1/24$	$hour^{-1}$	Estimated
$\boldsymbol{MAX\_MM\_DIV\_MSC}$	Maximum proliferation rate	$1/24$	$hour^{-1}$	Assumed
$\boldsymbol{MAX\_RESISTANT\_DIVISION\_RATE}$	Maximum proliferation rate	$1/48$	$hour^{-1}$	Estimated
$\boldsymbol{K_{MM}}$	Half-maximal concentration constant	$\sqrt{3} \cdot BDF_{thresh}$	$unitless$	*
$\boldsymbol{K_{MM\_MSC}}$	Half-maximal concentration constant	BDF_{basal}	$unitless$	*
$\boldsymbol{K_{R\_MM}}$	Half-maximal concentration constant	$\sqrt{1.25} \cdot BDF_{basal}$	$unitless$	*
$\boldsymbol{protect\_radius}$	Radius of MM birth/death advantage	20	μm	Assumed

	from MSC/preosteoblast			
$\boldsymbol{p\ resistance(p_{\Omega})}$	Resistance probability	$variable$	unitless	Assumed

- *Random motility coefficient of cell, MM_DiffCoef*: We assume that MM cells do not have random motility.
- *Chemotaxis coefficient, MM_TaxisCoef*: We assume that MM_TaxisCoef = MSC_TaxisCoef.
- *MM death rates, MM_DEATH, MM_EMDR_DEATH, MM_DEATH_BDF*: We assume that the lifespan of MM is similar to short-lived (3-5 days) and long-lived (several months) plasma cells[52]
- *Half maximal constant, K_{MM}*. We set $K_{MM} = \sqrt{3} \cdot BDF_{thresh}$, the value of BDF for which the myeloma proliferation rate is half maximum. This is estimated under the assumption that when $BDF = BDF_{thresh}$, i.e., when BDF is slightly above the basal level that is constantly present, the proliferation rate = 1/96 hour^{-1}. This value is based on the doubling time of myeloma cells *in vivo*.
- *Half maximal constant, K_{MM_MSC}*. We set $K_{MM\_MSC} = BDF_{basal}$, which is estimated under the assumption that when $BDF = BDF_{basal}$, i.e. in locations in the bone marrow that are not close to bone resorption, the myeloma proliferation rate = 1/48 hour^{-1} in the presence of MSC/preosteoblast (i.e. half the maximum proliferation rate).
- *Half maximal constant, K_{R_MM}*. We set $K_{R\_MM} = \sqrt{1.25} \cdot BDF_{basal}$, which is estimated so that when $BDF = BDF_{basal}$, the division rate of resistant myeloma cells is less than the division rate of sensitive myeloma cells and remains greater than the death rate in the absence of Bortezomib treatment.

Supplementary Table 6: BTZ Parameters

BTZ Parameter	Description	Value	Units	Source
$\boldsymbol{K_{dose}}$	Half-maximal concentration constant	0.05	$unitless$	Assumed

2. Biological Supplementary Methods

2.1. Cell culture

Human proteasome inhibitor sensitive (U266) myeloma cell and their proteasome inhibitor resistant derivatives (PSR[53, 54]) were a kind gift from Dr. Steven Grant at the University of Virginia, VA. U266 and PSR were transduced using QIAGEN lentiviral particles (CLS-

PCG-8 or CLS-PCR-8) according to manufacturer's instructions to express GFP and RFP respectively, generating U266-GFP and PSR-RFP. These cells were cultured in RPMI containing 10% FBS (PEAK), 1% penicillin-streptomycin. MCSF-generated macrophages were isolated from tibia and femur were harvested from 6-week-old C57Bl/6 RAG2-/- mice[55, 56]. Briefly,. Bone marrow cells were collected by centrifugation (10,000 g, 15s) cells were plated aMEM (+/+) containing 10% FBS (Peak), 1% penicillin-streptomycin and MCSF (300-25, Peprotech; 30ng/ml). Adherent macrophages were collected and used for downstream experiments after 72 hours. Murine mesenchymal stromal cells (MSCs) were isolated from tumor naive 4–6-week-old male and female C57/BL6 Rag2−/− mice[57]. Following removal of muscle tissue from the long bones, epiphyses were removed and bone marrow was depleted by centrifugation at 10,000g for 15 seconds. Flushed bones were then cut into 1–3 mm bone chips. The bone fragments were digested for 1 hour at 150 rpm, 37 °C in 1 mg/mL collagenase II (Invitrogen) in α-MEM with 15% FBS. The digested bone fragments were moved to 6-well tissue plates in 15% α-MEM, where the MSCs were allowed to migrate out of the bone chips and proliferate Human MSCs (PT-2501) and the murine preosteoblast cell line, MC3T3-E1 were purchased from Lonza and ATCC, respectively. Mouse MSCs and MC3T3-E1 cells were cultured in aMEM (-/-) containing 1% penicillin-streptomycin and 15 or 10% FBS (PEAK), respectively. Human MSCs were cultured as above except with 10% qualified FBS (Gibco).

2.2. MTT Assay

Myeloma cell lines, mouse MSCs or MC3T3-E1 were plated in 96-well plates at a density of 1×10^4 cells/well. Cells were treated with vehicle or a range of concentrations of zoledronate or bortezomib. Cell viability was assessed at 72 hours by the MTT assay following the manufacturer's instructions (CellTiter 96, #G3582, Pierce.) The absorbance was measured at 490nm after 3 hours of incubation at 37°C.

2.3. Osteoclastogenesis Assay

Osteoclast formation assays were performed using murine MCSF-generated macrophages as described in 2.1[55, 56]. Briefly, MCSF-generated macrophages from 4–6-week-old C57Bl/6 RAG2-/- mice were plated in a 96-well plate (25×10^3 cells/well) in

triplicate with MCSF (30ng/ml) and allowed to adhere for 24 hours. RANKL (315-11C, Peprotech) was given at the indicated doses with M-CSF on days 1 and 3. Cultures were fixed with 4% paraformaldehyde on day 5 and TRAcP stained. TRAcP positive multinucleated (>3) were deemed osteoclasts.

2.4. Osteoblast mineralization assay

Mouse MSCs, MC3T3-E1 cells or human MSCs (huMSCs) were differentiated with 1X StemXVivo mouse/rat or human osteogenic supplement (CCM009; R&D Systems) for indicated number of days. To assess the effect increased TGF-β has on OB differentiation, either exogenous human TGF-β1 (240-B; R&D systems) or vehicle (PBS) was added to cultures. To assess the effect decreased TGF-β levels have on OB differentiation, either anti-TGF-β antibody (1D11; R&D systems) or isotype control (13C4; R&D systems) was added to cultures. OB mineralization was assessed by Alizarin red staining[55, 56]. Formalin fixed osteoblast cultures were incubated in 40mM Alizarin red solution (pH 4.2) for 20 minutes at room temperature. Wells were washed thrice with distilled water for 5 minutes to remove excess stain, before air drying overnight. Images were captured on an EVOS Auto FL. The area covered by red stained mineral was quantified in Image J. Conditioned medium was generated from huMSCs or huMSCs differentiated for 7-28 days in osteogenic supplement by incubating cells in standard aMEM for 24hours.

2.5. MM-MSC and sensitive/resistant MM co-culture assays

Human MSCs (huMSCs; Lonza) were plated (2000 cells/100ul) or media alone in a 96 well plate and allowed to adhere overnight. The following day 50% of the media was removed and replaced with media containing U266-GFP+ MM cells in standard RPMI. Plates were centrifuged at 200g for 5 minutes to facilitate adhesion to MSCs. After 3 hours, bortezomib was added at indicated concentrations. Images were taken at 0 and 72 hours. The area covered by GFP+ MM cells was calculated in Fiji software[58]. U266-GFP and PSR-RFP (10,000 total cells/well,) were plated at indicated ratios in a 96-well plate with indicated concentrations of bortezomib. Cell confluency for GFP and RFP populations was measured every 12 hours using Incucyte S3. For long term (60 day and

30 day cultures). Cultures were set up as previously described. BTZ (10nM) was given over 72 hour periods and PI-sensitive U266 and PI-resistant PSR confluency was measured by fluorescent markers using the incucyte SX5. After treatment, media was replaced, after PBS washing, with standard RPMI. Images were taken at indicated timepoints with EVOS FL auto prior to media changes.

2.6. Flow cytometry

Left tibiae were used to assess tumor burden by GFP expression. Tibial ends were excised, whole bone marrow was isolated by centrifugation at 10,000g for 10 seconds. Red blood cells were lysed by RBC lysis buffer (R7757, Sigma-Aldrich) as per manufacturers guidelines. Bone marrow cells were subject to viability staining with Zombie Near-Infrared (NIR; 1:500; 423105, BioLegend). Appropriate compensation and fluorescence-minus-one (FMO) controls were generated in parallel either with aliquots of bone marrow cells or $U266^{GFP+/-}$. Stained controls and samples were analyzed using BD Biosciences LSRII flow cytometer. The percentage of GFP-expressing cells was gated on singlet live bone marrow cells in FCS Express 7. To address the proliferative advantages provided by cells of the BME to MM cells, U266 MM cells were stained with CM-DIL (Invitrogen; V22888) according to the manufacturer’s instruction and incubated with 50% (v/v) control, MSC, preosteoblasts (Day 7 or 14) or OB (day 21 or 28) conditioned media for 7 days, with media changes every 3 days. An aliquot of untreated CM-DIL+ cells was stained with Zombie NIR as above and used for baseline time point. At the end of the experiment, MM cells were stained with Zombie NIR for live/dead discrimination. The MFI for the CM-DiI channel was calculated at baseline and after 72 hours on live single cell cells using appropriate FMO controls. FCS Express 7 was used to calculate the proliferative index of these cells.

2.7. Microcomputed Tomography

Harvested right tibiae were fixed in 4% paraformaldehyde for 48 hours. Tibiae from mice from all time points were centralized and were subjected to micro-computed topography (μCT) scanning using SCANCO μ35 scanner to elucidate bone volume data at the proximal tibial metaphases. Individual bone scans were deidentified using numerical

codes during, and reidentified post analyses in a blinded fashion. Evaluation of trabecular bone microarchitecture was performed in a region that consisting of 1000 μm, beginning 500μm from the growth plate. A three-dimensional cubical voxel model of bone was built, and calculations were made for relative bone volume per total volume (BV/TV), trabecular number (Tb.N), trabecular thickness (Tb.Th), trabecular spacing (Tb.Sp) and connectivity density (Conn.D).

2.8. Immunofluorescence

Tibiae were decalcified in an excess of 10% EDTA for 21 days, refreshing EDTA every 48 hours before being placed in 30% sucrose (w/v) for 48 hours and subsequent cryosectioning. Tibia were mounted on Superfrost Plus slides (Fisher) with optimal cutting temperature (OCT) media and frozen on dry ice. Using a cryostat, 20 μm sections were made and stored at -80°C. For immunofluorescence, sections were kept on a slide warmer overnight at 56°C. Sections were rehydrated for 30 minutes at room temperature in PBS. Sections were blocked with 10% (v/v) normal goat serum and washed in PBS before staining addition of primary antibodies. Sections were incubated overnight at 4°C with primary rabbit antibodies at a dilution of 1:100 (anti-pHH3; 06-570 Millipore, anti-Osterix; ab22552, abcam: anti-aSMA: PA5-16697, Invitrogen,). Subsequently, slides were washed thrice in PBS and stained with goat anti-rabbit Alexa Fluor-647-conjugated secondary antibody (1:1000) at room temperature for 1h. DAPI (1ng/ml) was used as a nuclear counterstain. Mounted sections were imaged using 10X image tile-scans of whole tibia. The number of pHH3+ nuclei that co-localized with GFP+ cells were counted and the distance to nearest bone (tibia or cortical) was calculated. For MSC analyses, the percentage of total area in the trabecular region that was covered by αSMA+ cells were calculated on Fiji software. For preosteoblasts, the number of Osterix+ nuclei were counted and normalized to perimeter of trabecular bone for each mouse (Osx^+ cells/mm).

2.9. Histomorphometry TRAcP and ALP analyses

Additional tibia bone sections were cryosectioned and baked at 42°C overnight to improve tissue adhesion while retaining endogenous enzymatic activity. Tibial bone sections were rehydrated in PBS before incubation in napthol-AS-MX phosphate (Sigma-Aldrich; 855-

20) and fast blue RR salt (Sigma-Aldrich; FB25-10CAP) solution for 30 minutes to identify ALP+ regions with a dark blue stain. After washing in PBS, the same sections were incubated in basic stock solution for 5 minutes at 37°C. Tibial sections were developed in pararosaniline dye and sodium nitrite at 37°C for 3 minutes to highlight red TRAcP+ cells. Sections were further counterstained with hematoxylin to visualize bone morphology. Slides were mounted and imaged using EVOS Auto brightfield at 20X magnification. Five images per section were taken and the number of ALP+ cuboidal bone-lining osteoblasts and red multinucleated TRAcP+ osteoclasts per bone surface were calculated.

2.10 Ex vivo mathematical myeloma advisor (EMMA) platform: Pentecost Myeloma Research Center cohort. An *ex vivo* assay was used to quantify the chemosensitivity of primary MM cells[59-61]. CD138+ cells were isolated from fresh patient BM aspirate using Miltenyi (Bergisch Gladbach, Germany) 130-051-301 antibody-conjugated magnetic beads. MM cells (CD138$^+$) were seeded in collagen I coated Corning (Corning, NY) CellBIND 384 well plates and established human-derived stroma, containing approximately 4000 MM cells and 1000 stromal cells. Each well was filled with 80 µL of RPMI 1640 media supplemented with heat inactivated FBS penicillin/streptomycin, and patient-derived plasma (10%, freshly obtained from patient's own aspirate, filtered) and left overnight for adhesion of stroma. The following day, compounds were added using a robotic plate handler so that every drug/combination was tested at 5 (fixed concentration ratio, for combinations) concentrations (1:3 serial dilution) in two replicates. Negative controls (supplemented growth media with and without the vehicle control dimethyl sulfoxide [DMSO]) were included, as well as positive controls for each drug (cell line MM1.S at highest drug concentration). Plates were placed in a motorized stage microscope (EVOS Auto FL, Life Technologies, Carlsbad, CA) equipped with an incubator and maintained at 5% CO_2 and 37 °C. Each well was imaged every 30 min for a total duration of up to 6 days. A digital image analysis algorithm[59-61] was implemented to determine changes in viability of each well longitudinally across the 96 or 144hr intervals. This algorithm computes differences in sequential images and identifies live cells with continuous membrane deformations resulting from their interaction with the surrounding extracellular matrix. These interactions cease upon cell death. By applying this operation

to all 288 images acquired for each well, we quantified non-destructively, and without the need to separate the stroma and MM, the effect of drugs as a function of concentration and exposure time. Digital image analysis computes percent viability of MM cells for each time point and experimental condition (drug and concentration). For each patient-drug, we have a dose-time-response surface, which is abstracted into AUC (area under the curve), which is an area/integral measure of *ex vivo* response to therapy computed by taking an average of all *ex vivo* responses across all time (first 96h) and concentration. Clinical data was matched to patient samples ex vivo. Samples in the lowest AUC quartile were considered sensitive.

2.10. Statistical Analysis

Statistical analysis was performed using t-tests and the analysis of variance (ANOVA) with the appropriate post multiple comparison analysis indicated in figure legends in GraphPad prism 8.0-10.0.

Supplementary Figures and Legends

a

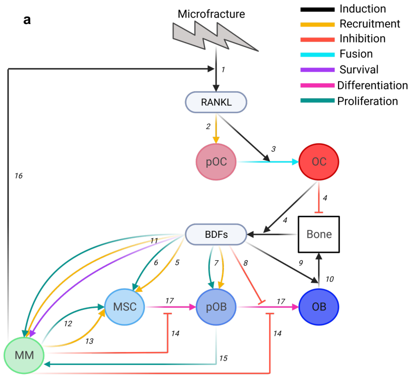

Line number	Experimental system and reference
1	Rat *in vivo* (PMID: 15704000), Mice *in vivo* (PMID: 21909105)
2	Human *in vitro* (PMID: 12958198); (PMID: 15248232)
3	Mouse *in vitro* (Supplementary Fig. 2f)
4	Mouse in vivo, human in vitro (PMID: 19584867)
5	Mouse in vivo, human in vitro (PMID: 19584867)
6	Mouse in vivo, human in vitro (PMID: 19584867)
7	Mouse in vivo, human in vitro (PMID: 19584867)
8	Mouse *in vitro* (Supplementary Fig. 2a)
9	Mouse in vivo (PMID: 22729283)
10	Mouse in vivo (PMID: 25079226)
11	Mouse and Human *in vitro* (Fig. 3f, Fig. 6j-m)
12	Mouse *in vivo* (Fig 5e)
13	Mouse in vitro and in vivo (PMID: 22102554)
14	Human (PMID: 15933061)
15	Human/mouse *in vitro* (Fig. 3f)
16	Human *in vitro* and in vivo (PMID: 11739153), Mouse *in vitro* and *in vivo* (PMID: 11562486)
17	Human *in vitro* (Fig. 3e) Mouse *in vitro* (Supplementary Fig. 3b and 4b)

b

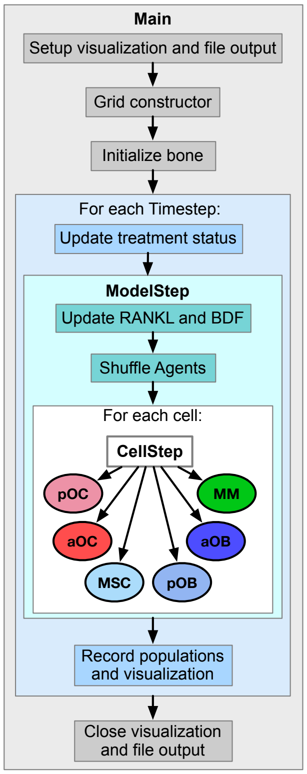

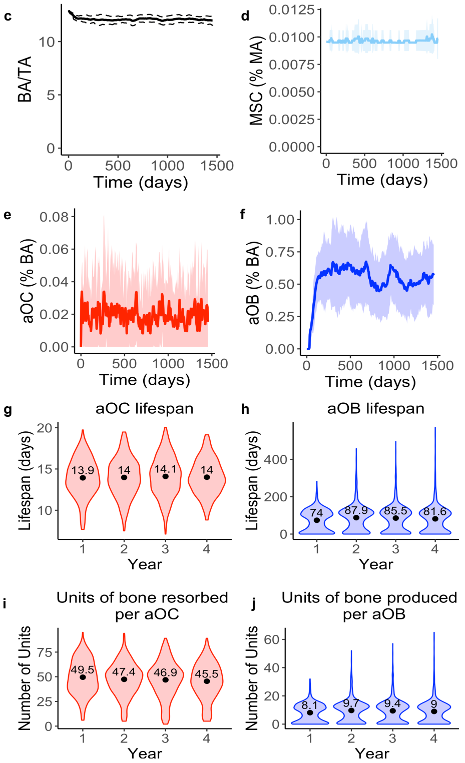

Supplementary Fig. 1. HCA captures key features of normal bone remodeling.

a. Interaction diagram (left) between cell types in the HCA and factors such as BDFs and RANKL (created with biorender.com). Table of references and experimental systems in which results were obtained (right).

b. HCA flow diagram adapted from Bravo et al. (2020)[6].

c. HCA model outputs of mean bone area to total area (BA/TA). Error bands represent standard deviation.

d. HCA model outputs of mean MSC content. Error bands represent standard deviation

e. HCA model outputs of mean osteoclast numbers. Error bands represent standard deviation

f. HCA model outputs of mean osteoblast numbers. Error bands represent standard deviation

g, Mean lifespan of osteoclasts and osteoblasts **(g)** over 4 years of normal bone homeostasis.

h, Mean lifespan of osteoblasts over 4 years of normal bone homeostasis

i, Mean units of bone resorbed per osteoclast each year of simulation.

j, Mean units of bone produced per osteoblast over course of the 4-year simulation.

Source data for **c-j** can be accessed at DOI 10.17605/OSF.IO/TNAX9.

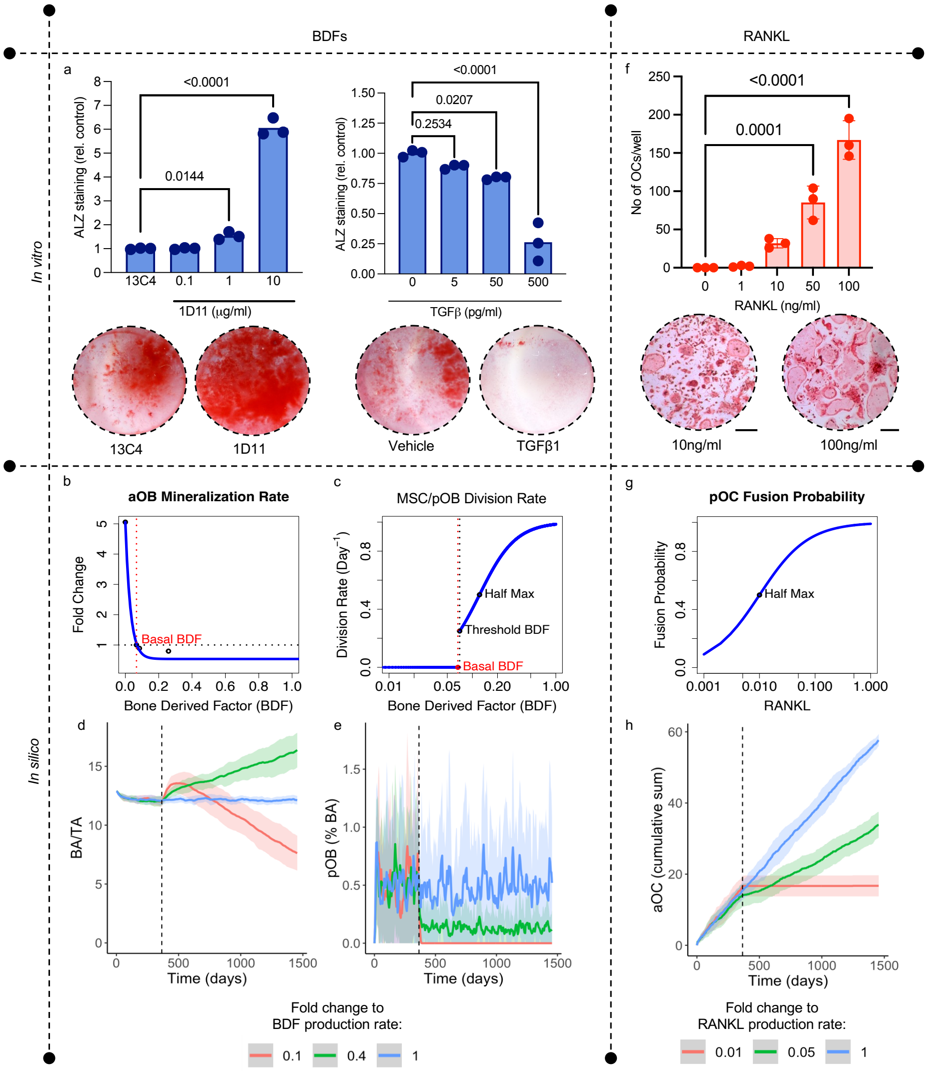

Supplementary Fig. 2. Inhibition of bone microenvironment cytokines in the HCA. a, The preosteoblast cell line MC3T3-E1 was cultured in osteogenic supplement with indicated concentrations of either isotype control antibody 13C4 or anti-TGF-β neutralizing antibody 1D11 (left) or TGF-β1 (right) for 14 days. Alizarin red was used to identify mineralized area. Results are normalized relative to isotype/vehicle-treated controls. Representative images of alizarin staining from control and highest concentrations (below; red staining). N=3 individual experiments per group. **b-c**, Plots of

functional forms used to represent the fold change to osteoblast mineralization rate (**b**) and MSC/preosteoblast division rate (**c**). The black dots (**b**) represent the data from the *in vitro* experiments with the MC3T3-E1 cells that were used to fit the exponential decay function, as described in section 1.1 of supplementary methods. The red dot (**c**) represents the lack of MSC/preosteoblast division rate when BDF is at a basal level (dotted red line), and the black dot (**c**) represents the division rate when BDF is at the threshold level for proliferation (dotted black line). Another black dot highlights the half maximum division rate defined by the Hill function. **d-e,** Computational outputs of bone area to total area ratio (d) and number of pOBs per unit of bone (e) when bone-derived factors are inhibited, with standard deviation shown (shaded colors; n = 25). f, Mean number of of TRAcP+ multinucleated OC per well produce by addition of indicated concentration of RANKL to murine MCS-F generated macrophages for 6 days. Representative images of TRAcP+ osteoclasts, scale bar = 200 microns (below) g, Plot of functional form used to represent the probability of pOC fusion. h, Computational outputs of cumulative number of aOCs following RANKL inhibition, with standard deviation shown (shaded colors; n = 25) (g). BDF or RANKL inhibition begins at day 365 (black dotted line) and is applied continuously.

Data are mean ± SD
Statistical significance was determined by one-way ANOVA with a Dunnet's multiple comparison test (**a** and **f**). Source data are provided as a Source Data file for **a** and **f.** Source data for **b-e** and **g-h** can be accessed at DOI 10.17605/OSF.IO/TNAX9.

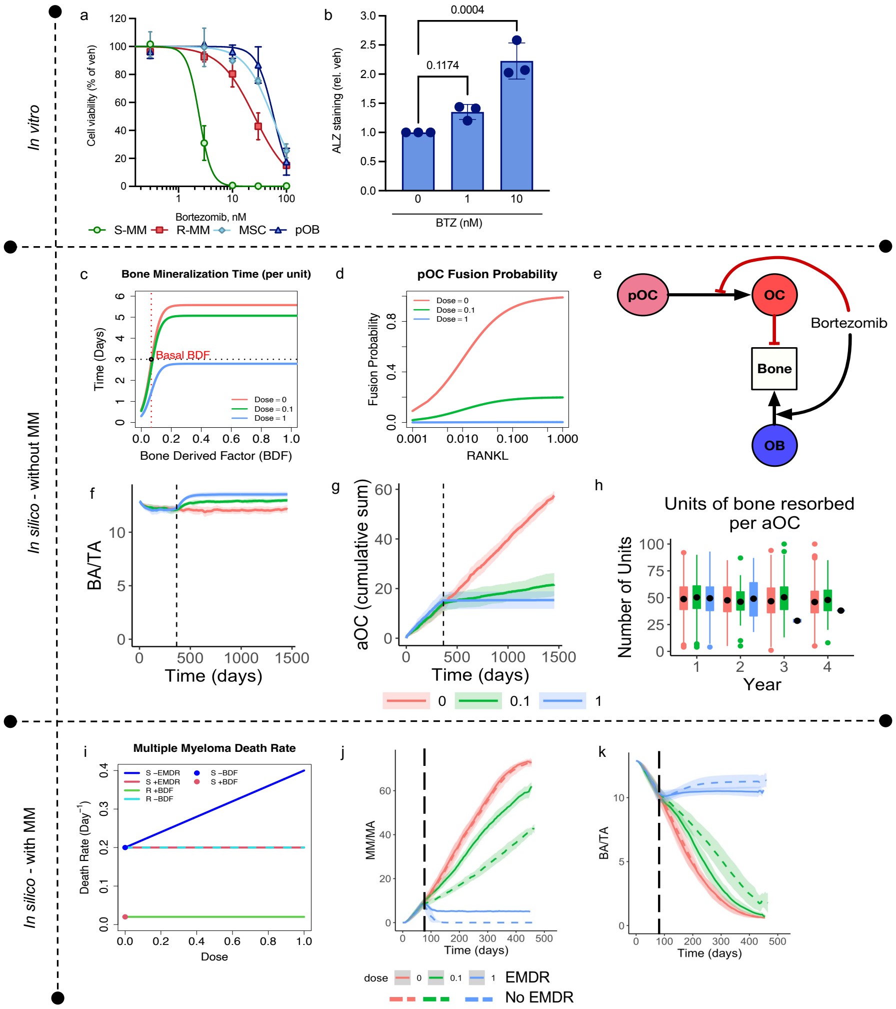

Supplementary Fig. 3. The impact of bortezomib (BTZ) on cells of the myeloma bone microenvironment *in vitro* and *in silico*. **a**, Sensitive U266 (S-MM, n=3 individual experiments) and resistant PSR (R-MM) myeloma, murine mesenchymal stromal cells (MSCs) and the preosteoblast cell line MC3T3-E1 (preosteoblast) were treated with varying concentrations of BTZ. Viability was measured after 72 hours by MTT assay. Results are normalized as percentage of control. Mean ± SD. **b**, the preosteoblast cell line MC3T3-E1 were cultured in osteogenic supplement with indicated concentrations of BTZ. Alizarin red was used to identify mineralized area. Results are normalized to vehicle-treated controls. Mean ± SD **c-d**, Plots of functional forms used to represent the osteoblast mineralization time (**c**) and the probability of preosteoclast fusion (**d**) with indicated concentrations of bortezomib. **e**, Interaction diagram of normal bone cells

with bortezomib. Red line denotes inhibition, black like indicates stimulation. **f-h**, Computational outputs of bone area to total area ratio (**f**), cumulative number of osteoclasts (**g**), and units of bone resorbed per osteoclast (**h**) following treatment of normal bone model with indicated concentrations of bortezomib **(h;** centre line at the median, upper bound at 75th percentile, lower bound at 25th percentile with whiskers that extend to the minimum and maximum values within 1.5 times the interquartile range and any data points outside this range are plotted individually). Treatment begins on day 365 (black dotted line) and is applied continuously for 3 years. **i**, Plot showing S-MM and R-MM death rate as a function of bortezomib with/without (+/-) BDF and +/- EMDR. **j**, Model outputs of MM growth with high and low doses of bortezomib with/without EMDR. Treatment begins when MM/MA=10% (black dotted line) and is applied continuously until MM/MA=20%. **k**, Flow chart outlining decisions MM cells make under BTZ treatment.

Data are mean ± SD Statistical significance was determined by one-way ANOVA with a Dunnet's multiple comparison test (**b**). Source data are provided as a Source Data file for **a** and **b.** Source data for **c-k** can be accessed at DOI 10.17605/OSF.IO/TNAX9.

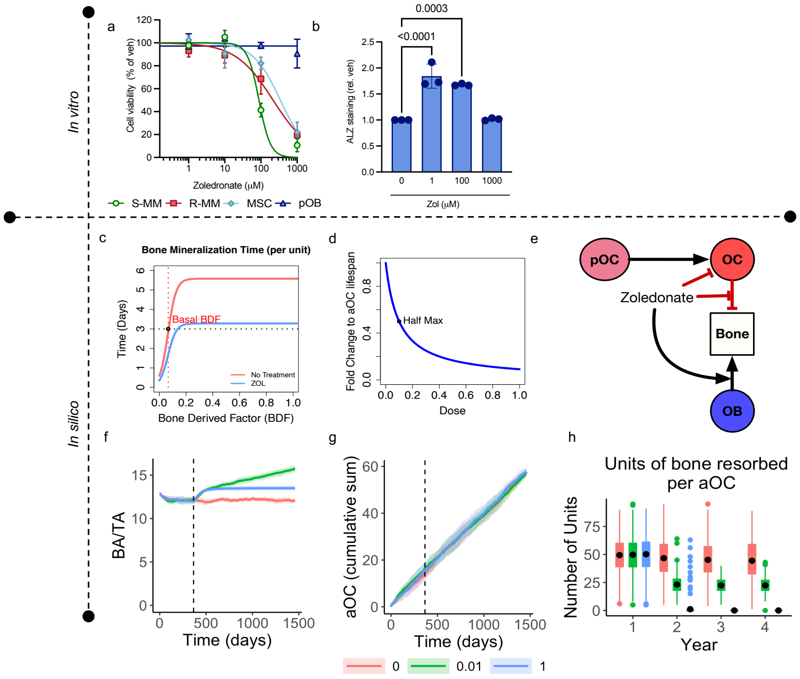

Supplementary Fig. 4. The impact of zoledronate (ZOL) on cells of the myeloma bone microenvironment *in vitro* and in *silico*. **a**, Sensitive U266 (S-MM, n=3 individual experiments) and resistant PSR (R-MM) myeloma, murine mesenchymal stromal cells (MSCs) and the preosteoblast cell line MC3T3-E1 (preosteoblast) were treated with varying concentrations of zoledronate (ZOL). Viability was measured after 72 hours by MTT assay. Results are normalized as percentage of control. **b**, The preosteoblast cell line MC3T3-E1 were cultured in osteogenic supplement with indicated concentrations of ZOL. Alizarin red was used to identify mineralized area. Results are normalized to vehicle-treated controls. Data are mean ± SD. **c-d**, Plots of functional forms used to represent the osteoblast mineralization time (**c**) and the fold change to osteoclast lifespan (**d**) with indicated concentrations of ZOL. **e**, Interaction diagram of normal bone cells with ZOL. Red line denotes inhibition, black like indicates stimulation. **f-h**, Computational outputs of bone area to total area ratio (**f**), cumulative number of osteoclasts (**g**), and units of bone resorbed per osteoclast (**h;** centre line at the median, upper bound at 75th percentile, lower bound at 25th percentile with whiskers that extend to the minimum and maximum values within 1.5 times the interquartile range and any data points outside this

range are plotted individually) following treatment of normal bone model with indicated concentrations of ZOL. Treatment begins on day 365 (black dotted line) and is applied continuously for 3 years. Statistical significance was determined by one-way ANOVA with a Dunnet's multiple comparison test (**b**). Source data are provided as a Source Data file for **a** and **b.** Source data for **c-h** can be accessed at DOI 10.17605/OSF.IO/TNAX9.

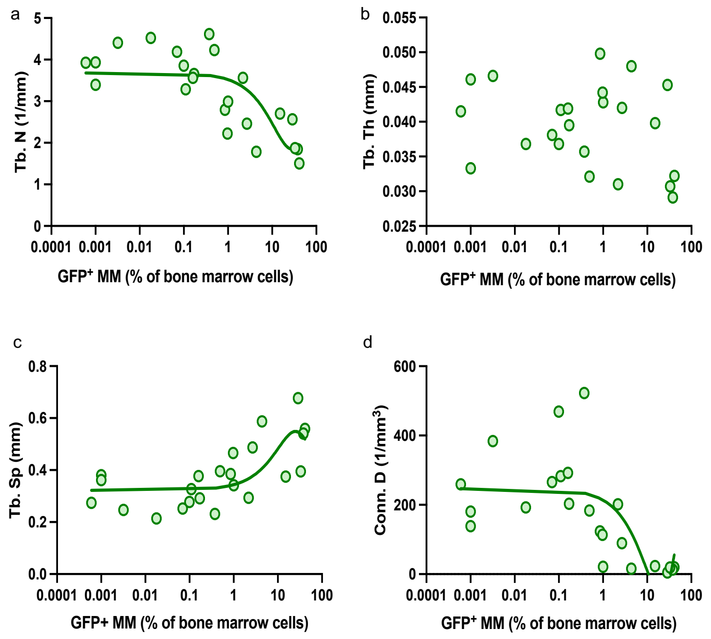

Supplementary Fig. 5. Myeloma alters bone microarchitecture *in vivo*. a-d, Tumor burden in tibia and femur was assessed by GFP positivity by flow cytometry in U266-bearing mice (n=23 individual tumor-bearing tibiae). High resolution microCT was used to assess trabecular number (Tb.N; **a**), thickness (Tb.Th; **b**) and spacing (Tb.Sp; **c**) and degree of connectivity (Conn.D; **d**). Source data are provided as a Source Data file for **a-d.**

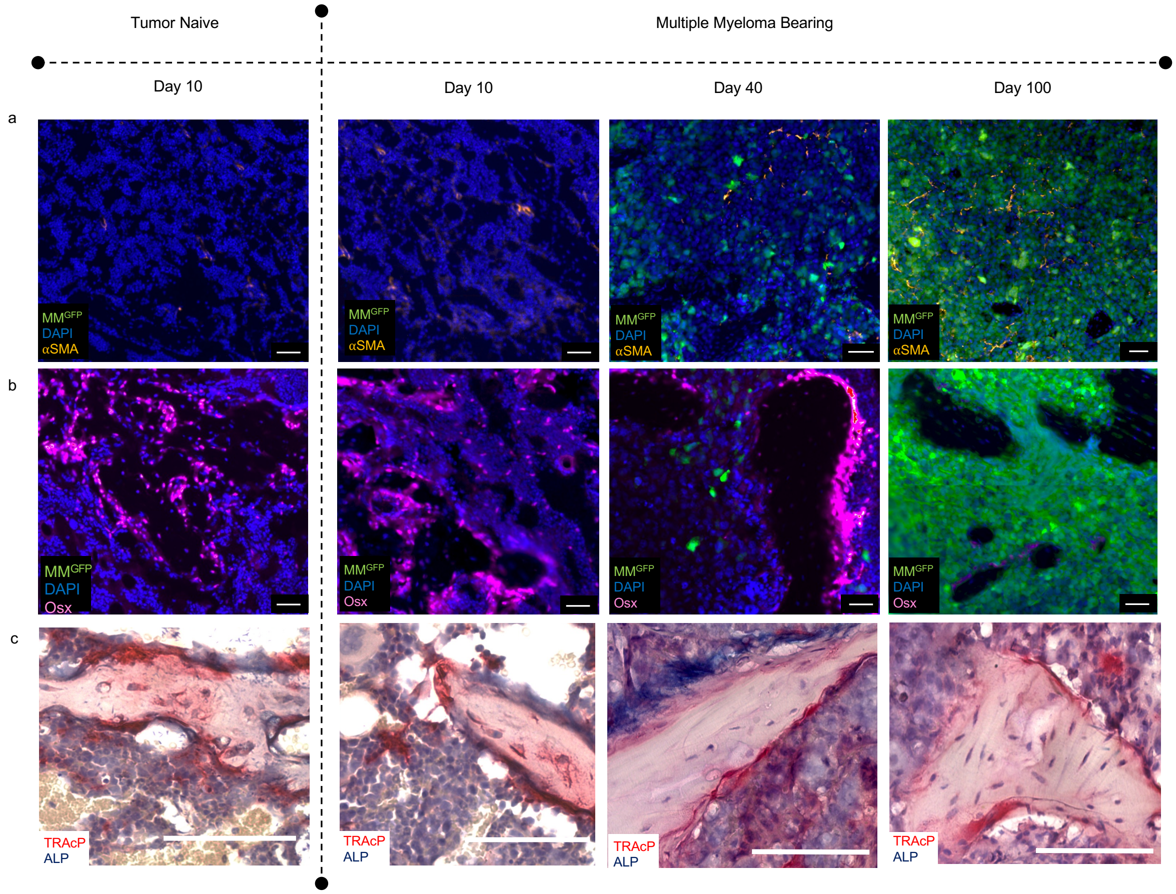

Supplementary Fig. 6. Multiple myeloma alters bone marrow microenvironment. a-b, Tumor naïve or U266GFP-bearing mice were sacrificed on indicated days to identify and localize αSMA+ mesenchymal stromal cells (gold; **a**), osterix+ preosteoblasts (pink; **b**) and GFP+ U266 cells (green; **a-b**) by immunofluorescence. Scale bars = 50μm **c**, Brightfield images of TRAcP+ multinucleated osteoclasts (red) and ALP+ cuboidal bone-lining osteoblasts (blue). Scale bars = 100μm. Images correspond with quantifications from Figure 6.

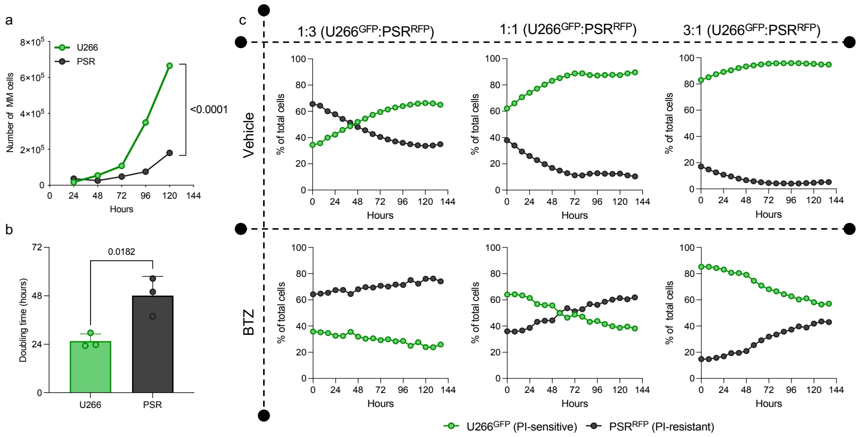

Supplementary Fig. 7. PI-sensitive MM cells outgrow their PI-resistant counterparts under normal growth conditions. **a-b**, PI-sensitive U266 and PI-resistant PSR MM cell lines were grown in standard culture conditions, the number of cell number was recorded using trypan blue staining over 120h (**a,** mean±SD, n=3 individual experiments per cell line) and used to calculate doubling times (**b,** mean±SD, n=3 individual experiments per cell line). Proteasome inhibitor sensitive GFP expressing U266 MM cells were incubated at indicated ratios with their proteasome inhibitor RFP expressing counterparts (PSR). The percentage of each population under vehicle control conditions was monitored over time using real time imaging microscopy (Incucyte). In parallel the impact of BTZ (4nM) on the outgrowth of the populations was observed revealing the emergence of the resistant population over time. Statistical significance was determined by two-way ANOVA with a Šídák's multiple comparison (**a**) or an unpaired t test (**b**). Source data are provided as a Source Data file for **a**-**c.**

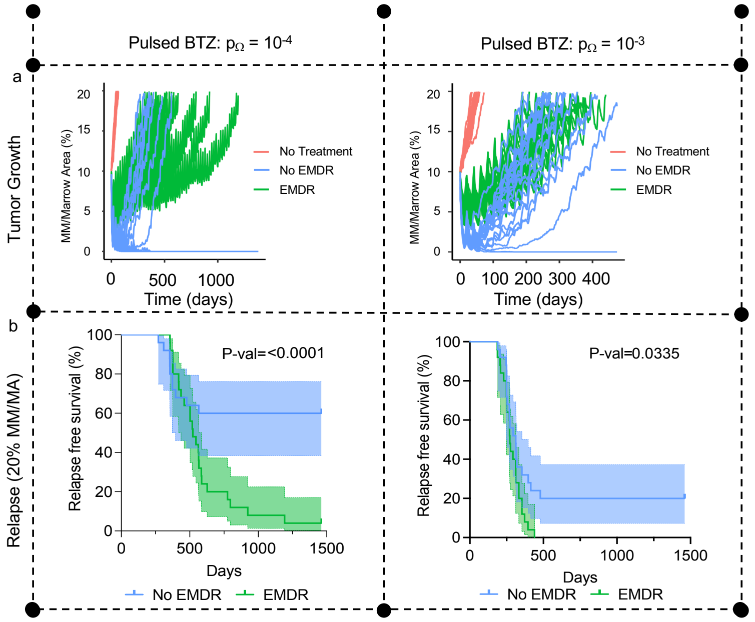

Supplementary Fig. 8. EMDR increases rate of myeloma relapse and tumor heterogeneity under pulsed bortezomib treatment. Pulsed BTZ treatment (2 weeks on therapy, 1 week off) was applied to HCA model when MM burden reached 10% of the marrow. **a-b**, Tumor growth (**a**) for individual simulations (n = 25 per condition) and Kaplan Meier plots of rate of relapse (**b**) for pulsed BTZ therapy were calculated when $p_\Omega = 10^{-4}$ (left column) and $p_\Omega = 10^{-3}$ (right column). Error bands represent 95% confidence intervals. Statistical significance was determined by and log-rank tests (b). Source data for **a-b** can be accessed at DOI 10.17605/OSF.IO/TNAX9.

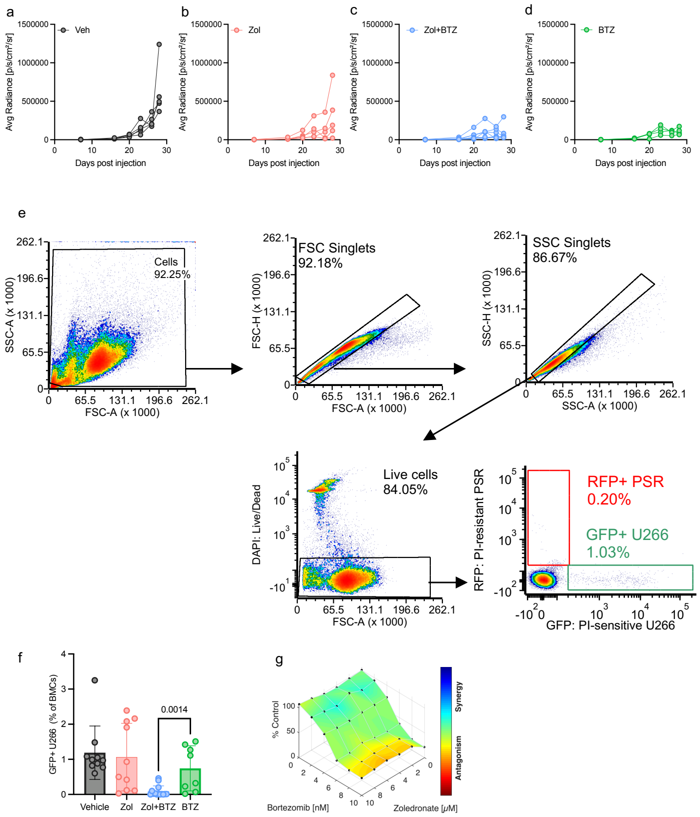

Supplementary Fig. 9. Combination of zoledronate and bortezomib reduces U266 growth in vivo. a-d U266-Luc growth by BLI after MM cells (90% U266-GFP$^+$Luc$^{+,}$ 10% PSR-RFP) were tail vein injected into NSG mice. Mice were divided into two groups and pre-treated with vehicle or Zol (30 μg/kg) for 1 week prior to mice being randomized and treated with either vehicle (n=5 mice, a), Zol (n=5 mice, b), Zol+BTZ (n=7 mice, c), or BTZ (0.5mg/kg; n= 4 mice, d). Each line indicates individual mice. Each dot represents indicated time point post tumor cell inoculation. Related to Figure 6. **e**, Example gating strategy for the analysis of GFP+ and RFP+ multiple myeloma cells. Related to main figure 7e-f and supplementary figure 9f. **f,** The average number (mean± SD) of GFP+ U266 MM cells as a percentage of live (DAPI negative) bone marrow cells in mice treated with either vehicle (n=10 femurs), Zol (n=10 femurs), Zol+BTZ (n=14 femurs) or BTZ (n=8 femurs). Each dot represents an individual tumor from a femur. **g**, U266 myeloma cells were treated with the indicated concentration of zoledronate and/or bortezomib for 72 hours. MTT assay was performed and the mean number of viable myeloma cells as a percentage of vehicle treated control was calculated. Plot shows LOEWE synergy

mapped to dose-response matrix. Green areas indicate concentrations at which no synergy or antagonism was observed. Yellow indicates mild antagonism. Statistical significance was determined by unpaired t test (**f**). Source data are provided as a Source Data file for **a**-**d, f-g.**

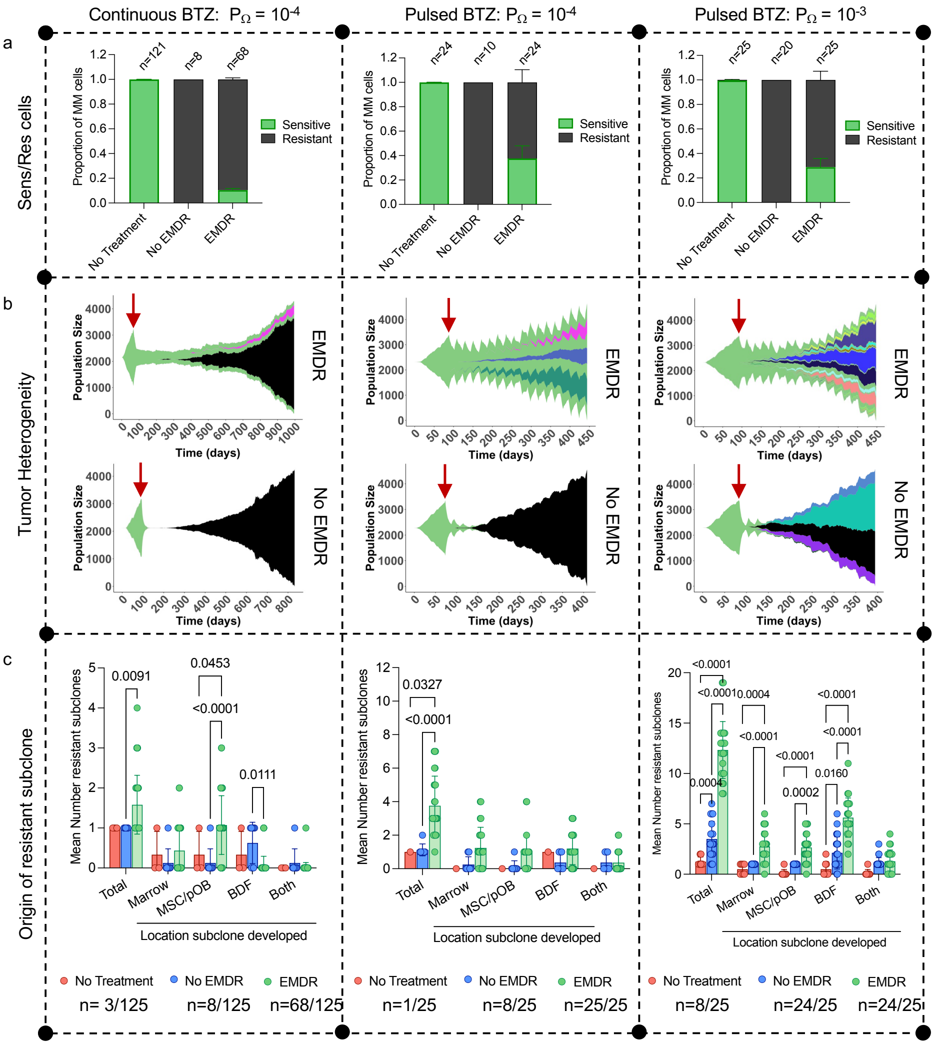

Supplementary Fig. 10. EMDR increases tumor heterogeneity under continuous and pulsed bortezomib treatment. Continuous BTZ treatment (left column) or Pulsed BTZ treatment (2 weeks on therapy, 1 week off; middle and right column) were applied to HCA model when MM burden reached 10% of the marrow. **a-c**, The mean ± SD proportion of sensitive/resistant MM cells at end point (MM = 20% of the marrow; **a**) and tumor heterogeneity (**b-c**) were assessed with two resistance probabilities (left

and middle columns, $p_\Omega = 10^{-4}$, right column $p_\Omega = 10^{-3}$) in the presence or absence of EMDR. The n numbers for each group are displayed above each figure as the number of simulations that developed resistant clones. **b**, Muller plots indicating resistant subclones arising in tumor, created using EvoFreq[62]. Each color represents a unique subclone. Red arrow indicates start of treatment. **c**, The number of resistant sub-clones (mean± SD) arising in tumors that reached 20% and contained resistant subclones (MM/MA) following continuous or pulsed BTZ treatment at different locations within the BME with/without EMDR. 'Both' refers to resistant subclones that arise close to MSCs and BDFs. The n numbers for each group are displayed under each figure as the number of simulations that developed resistant clones/total number of simulations ran Overall, EMDR leads to significantly higher numbers of resistant subclones. Statistical significance was determined by two-way ANOVA with a Tukey's multiple comparison test. Source data for **a-c** can be accessed at DOI 10.17605/OSF.IO/TNAX9.

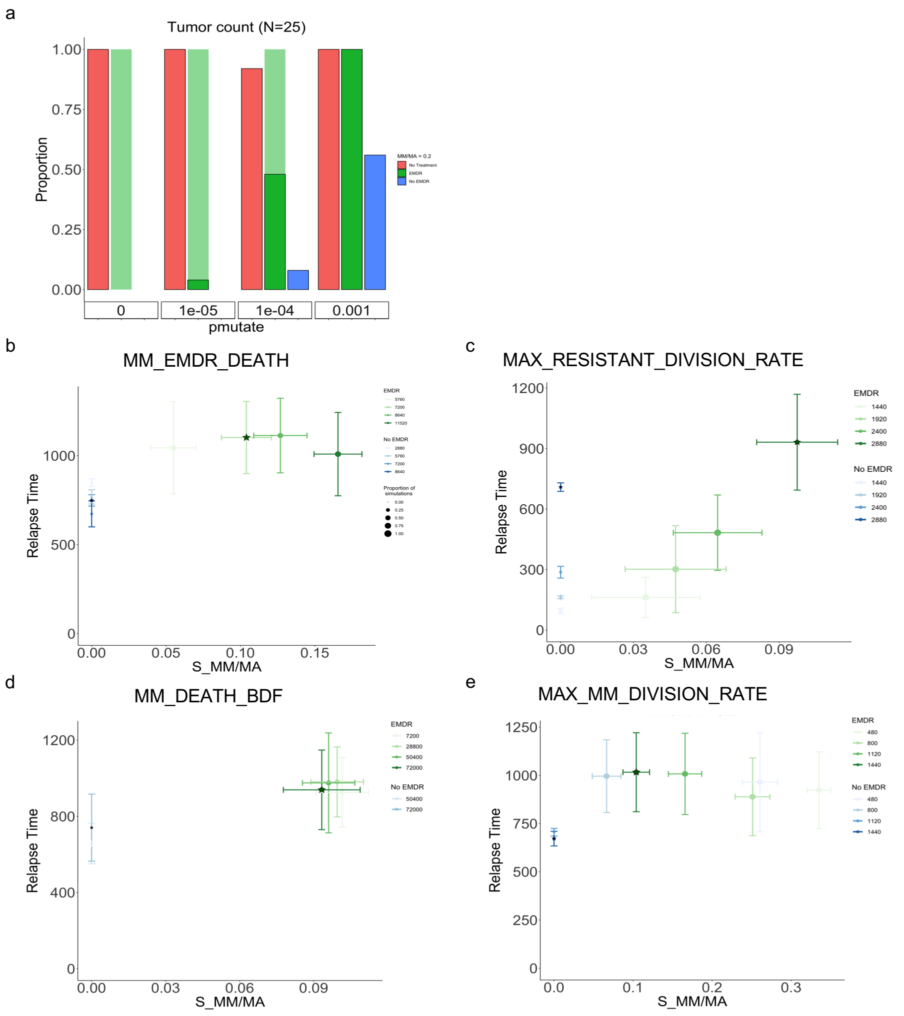

Supplementary Fig. 11. The impact of select parameter values on key model outputs. Continuous BTZ treatment was applied to HCA model when MM burden reached 10% of the marrow. **a.** Proportion of tumors that survived (lighter shade) or relapsed (darker shade) was assessed as resistance probability increased. **b-e**. Select parameters were varied to assess how the relapse time (when MM burden reached 20% of the marrow) and proportion of sensitive cells at endpoint changed due to parameter values ($p_{\Omega} = 10^{-4}$). MM_EMDR_DEATH is the lifespan of sensitive cells (+ BTZ) protected by EMDR (**b**). MAX_RESISTANT_DIVISION_RATE is the maximum division time of

resistant cells (+/- BTZ) (**c**). MM_DEATH_BDF is the lifespan of sensitive cells (-BTZ) or resistant cells (+/- BTZ) in the presence of BDF (**d**). MAX_MM_DIVISION_RATE is the maximum division time of sensitive cells (+/- BTZ) in the presence of MSC/preosteoblast (**e**). The star marker indicates the outputs corresponding to the baseline parameter value. The circular marker indicates the mean value of the outputs with error bars representing the standard deviation of the simulations (n = 25); the size of the circular marker increases as the proportion of simulations that reached the endpoint increased. Source data for **a-e** can be accessed at DOI 10.17605/OSF.IO/TNAX9.

Supplementary References:

1. Araujo A, Cook LM, Lynch CC, Basanta D. An integrated computational model of the bone microenvironment in bone-metastatic prostate cancer. *Cancer Res* 2014 May 1; **74**(9)**:** 2391-2401.

2. Araujo A, Cook LM, Lynch CC, Basanta D. Size Matters: Metastatic cluster size and stromal recruitment in the establishment of successful prostate cancer to bone metastases. *Bull Math Biol* 2018 May; **80**(5)**:** 1046-1058.

3. Cook LM, Araujo A, Pow-Sang JM, Budzevich MM, Basanta D, Lynch CC. Predictive computational modeling to define effective treatment strategies for bone metastatic prostate cancer. *Sci Rep* 2016 Jul 14; **6:** 29384.

4. Terstappen LW, Levin J. Bone marrow cell differential counts obtained by multidimensional flow cytometry. *Blood Cells* 1992; **18**(2)**:** 311-330; discussion 331-312.

5. Pittenger MF, , Mackay AM, , Beck SC, *, et al.* Multilineage potential of adult human mesenchymal stem cells. 1999. p. 143-147.

6. Bravo RR, Baratchart E, West J, Schenck RO, Miller AK, Gallaher J*, et al.* Hybrid Automata Library: A flexible platform for hybrid modeling with real-time visualization. *PLoS Comput Biol* 2020 Mar; **16**(3)**:** e1007635.

7. Sims NA, , Martin TJ, . Coupling the activities of bone formation and resorption: a multitude of signals within the basic multicellular unit. 2014. p. 1-10.

8. Kular J, , Tickner J, , Chim SM, *, et al.* An overview of the regulation of bone remodelling at the cellular level. The Canadian Society of Clinical Chemists; 2012. p. 863-873.

9. Manolagas SC, . Birth and death of bone cells: Basic regulatory mechanisms and implications for the pathogenesis and treatment of osteoporosis. 2000. p. 115-137.

10. Roodman GD, . Osteoclast differentiation. 1991. p. 389-409.

11. Alon U, . An introduction to systems biology: design principles of biological circuits. Chapman and Hall/CRC; 2006.

12. Mosheimer BA, , Kaneider NC, , Feistritzer C, *, et al.* Expression and function of RANK in human monocyte chemotaxis. 2004. p. 2309-2316.

13. Breuil V, , Schmid-Antomarchi H, , Schmid-Alliana A, *, et al.* The receptor activator of nuclear factor (NF)-kappaB ligand (RANKL) is a new chemotactic factor for human monocytes.; 2003. p. 1751-1753.

14. Anderson ARA, . A hybrid mathematical model of solid tumour invasion: The importance of cell adhesion. 2005. p. 163-186.

15. Delaisse J-M, . The reversal phase of the bone-remodeling cycle: cellular prerequisites for coupling resorption and formation. Nature Publishing Group; 2014. p. 1-8.

16. Sims NA, , Martin TJ, . Osteoclasts Provide Coupling Signals to Osteoblast Lineage Cells Through Multiple Mechanisms. 2020. p. 507-529.

17. Novack DV, Faccio R. Osteoclast motility: putting the brakes on bone resorption. *Ageing Res Rev* 2011 Jan; **10**(1)**:** 54-61.

18. van den Dries K, Linder S, Maridonneau-Parini I, Poincloux R. Probing the mechanical landscape - new insights into podosome architecture and mechanics. *J Cell Sci* 2019 Dec 13; **132**(24).

19. Bianco P, Cao X, Frenette PS, Mao JJ, Robey PG, Simmons PJ*, et al.* The meaning, the sense and the significance: translating the science of mesenchymal stem cells into medicine. *Nat Med* 2013 Jan; **19**(1)**:** 35-42.

20. Greenbaum A, Hsu YM, Day RB, Schuettpelz LG, Christopher MJ, Borgerding JN*, et al.* CXCL12 in early mesenchymal progenitors is required for haematopoietic stem-cell maintenance. *Nature* 2013 Mar 14; **495**(7440)**:** 227-230.

21. Mendez-Ferrer S, Michurina TV, Ferraro F, Mazloom AR, Macarthur BD, Lira SA*, et al.* Mesenchymal and haematopoietic stem cells form a unique bone marrow niche. *Nature* 2010 Aug 12; **466**(7308)**:** 829-834.

22. Tang Y, Wu X, Lei W, Pang L, Wan C, Shi Z*, et al.* TGF-beta1-induced migration of bone mesenchymal stem cells couples bone resorption with formation. *Nat Med* 2009 Jul; **15**(7)**:** 757-765.

23. Wu M, , Chen G, , Li YP, . TGF-β and BMP signaling in osteoblast, skeletal development, and bone formation, homeostasis and disease. 2016.

24. Civitelli R. Cell-cell communication in the osteoblast/osteocyte lineage. *Arch Biochem Biophys* 2008 May 15; **473**(2)**:** 188-192.

25. Stains JP, Civitelli R. Cell-cell interactions in regulating osteogenesis and osteoblast function. *Birth Defects Res C Embryo Today* 2005 Mar; **75**(1)**:** 72-80.

26. Stains JP, Watkins MP, Grimston SK, Hebert C, Civitelli R. Molecular mechanisms of osteoblast/osteocyte regulation by connexin43. *Calcif Tissue Int* 2014 Jan; **94**(1)**:** 55-67.

27. Watkins M, Grimston SK, Norris JY, Guillotin B, Shaw A, Beniash E*, et al.* Osteoblast connexin43 modulates skeletal architecture by regulating both arms of bone remodeling. *Mol Biol Cell* 2011 Apr 15; **22**(8)**:** 1240-1251.

28. Nakashima T, , Hayashi M, , Fukunaga T, *, et al.* Evidence for osteocyte regulation of bone homeostasis through RANKL expression. 2011.

29. Xiong J, , Onal M, , Jilka RL, *, et al.* Matrix-embedded cells control osteoclast formation. 2011. p. 1235-1241.

30. Batlle E, , Massagué J, . Transforming Growth Factor-β Signaling in Immunity and Cancer. 2019. p. 924-940.

31. Sezer O, , Heider U, , Zavrski I, *, et al.* RANK ligand and osteoprotegerin in myeloma bone disease. 2003. p. 2094-2098.

32. Xu S, , Menu E, , De Becker A, *, et al.* Bone marrow-derived mesenchymal stromal cells are attracted by multiple myeloma cell-produced chemokine CCL25 and favor myeloma cell growth in vitro and in vivo. 2012. p. 266-279.

33. Hideshima T, , Mitsiades C, , Tonon G, *, et al.* Understanding multiple myeloma pathogenesis in the bone marrow to identify new therapeutic targets. 2007. p. 585-598.

34. Edwards CM, , Zhuang J, , Mundy GR, . The pathogenesis of the bone disease of multiple myeloma. 2008. p. 1007-1013.

35. Kassen D, Lath D, Lach A, Evans H, Chantry A, Rabin N*, et al.* Myeloma impairs mature osteoblast function but causes early expansion of osteo-progenitors: temporal changes in bone physiology and gene expression in the KMS12BM model. *Br J Haematol* 2016 Jan; **172**(1)**:** 64-79.

36. Hurchla MA, , Garcia-Gomez A, , Hornick MC, *, et al.* The epoxyketone-based proteasome inhibitors carfilzomib and orally bioavailable oprozomib have anti-resorptive and bone-anabolic activity in addition to anti-myeloma effects. 2013. p. 430-440.

37. Meads MB, , Gatenby RA, , Dalton WS, . Environment-mediated drug resistance: A major contributor to minimal residual disease. 2009. p. 665-674.

38. Janssens K, ten Dijke P, Janssens S, Van Hul W. Transforming growth factor-beta1 to the bone. *Endocr Rev* 2005 Oct; **26**(6)**:** 743-774.

39. Brown DR, . Dependence of Neurones on Astrocytes in a Coculture System Renders Neurones Sensitive to Transforming Growth Factor\gb\1-Induced Glutamate Toxicity. Wiley Online Library; 1999. p. 943-953.

40. Goodhill GJ, . Diffusion in axon guidance. Wiley Online Library; 1997. p. 1414-1421.

41. Zaccai NR, , Serdyuk IN, , Zaccai J, . Methods in molecular biophysics: structure, dynamics, function for biology and medicine. Cambridge University Press; 2017.

42. Syková E, , Nicholson C, . Diffusion in brain extracellular space. American Physiological Society; 2008. p. 1277-1340.

43. Kaminska B, , Wesolowska A, , Danilkiewicz M, . TGF beta signalling and its role in tumour pathogenesis.; 2005. p. 329-337.

44. Helgason B, , Perilli E, , Schileo E, *, et al.* Mathematical relationships between bone density and mechanical properties: A literature review. 2008. p. 135-146.

45. Pfeilschifter J, , Diel I, , Scheppach B, *, et al.* Concentration of transforming growth factor beta in human bone tissue: relationship to age, menopause, bone turnover, and bone volume.; 1998. p. 716-730.

46. Parfitt AM, . Osteonal and hemi-osteonal remodeling: The spatial and temporal framework for signal traffic in adult human bone. 1994. p. 273-286.

47. Akchurin T, , Aissiou T, , Kemeny N, *, et al.* Complex dynamics of osteoclast formation and death in long-term cultures. 2008.

48. Wergedal J, , Stauffer M, , Baylink D, *, et al.* Inhibition of bone matrix formation, mineralization, and resorption in thyroparathyroidectomized rats.; 1973. p. 1052-1058.

49. Bloebaum RD, , Bachus KN, , Momberger NG, *, et al.* Mineral apposition rates of human cancellous bone at the interface of porous coated implants. 1994. p. 537-544.

50. Tranquillo RT, , Zigmond SH, , Lauffenburger DA, . Measurement of the chemotaxis coefficient for human neutrophils in the under-agarose migration assay. 1988. p. 1-15.

51. Bianchi A, , Painter KJ, , Sherratt JA, . Spatio-temporal Models of Lymphangiogenesis in Wound Healing. Springer US; 2016. p. 1904-1941.

52. Khodadadi L, , Cheng Q, , Radbruch A, *, et al.* The Maintenance of Memory Plasma Cells.; 2019. p. 721.

53. Chen S, , Dai Y, , Pei X-Y, *, et al.* CDK Inhibitors Upregulate BH3-Only Proteins to Sensitize Human Myeloma Cells to BH3 Mimetic TherapiesBH3-Only Proteins Mediate BH3 Mimetic Activity. AACR; 2012. p. 4225-4237.

54. Chen S, , Zhang Y, , Zhou L, *, et al.* A Bim-targeting strategy overcomes adaptive bortezomib resistance in myeloma through a novel link between autophagy and apoptosis. American Society of Hematology Washington, DC; 2014. p. 2687-2697.

55. Bishop RT, Marino S, de Ridder D, Allen RJ, Lefley DV, Sims AH*, et al.* Pharmacological inhibition of the IKKepsilon/TBK-1 axis potentiates the anti-tumour and anti-metastatic effects of Docetaxel in mouse models of breast cancer. *Cancer Lett* 2019 May 28; **450:** 76-87.

56. Marino S, Bishop RT, de Ridder D, Delgado-Calle J, Reagan MR. 2D and 3D In Vitro Co-Culture for Cancer and Bone Cell Interaction Studies. *Methods Mol Biol* 2019; **1914:** 71-98.

57. McGuire JJ, Frieling JS, Lo CH, Li T, Muhammad A, Lawrence HR*, et al.* Mesenchymal stem cell-derived interleukin-28 drives the selection of apoptosis resistant bone metastatic prostate cancer. *Nat Commun* 2021 02; **12**(1)**:** 723.

58. Schindelin J, , Arganda-Carreras I, , Frise E, *, et al.* Fiji: an open-source platform for biological-image analysis. Nature Publishing Group; 2012. p. 676-682.

59. Silva A, Jacobson T, Meads M, Distler A, Shain K. An Organotypic High Throughput System for Characterization of Drug Sensitivity of Primary Multiple Myeloma Cells. *J Vis Exp* 2015 Jul 15; (101)**:** e53070.

60. Silva A, Silva MC, Sudalagunta P, Distler A, Jacobson T, Collins A*, et al.* An Ex Vivo Platform for the Prediction of Clinical Response in Multiple Myeloma. *Cancer Res* 2017 Jun 15; **77**(12)**:** 3336-3351.

61. Sudalagunta P, Silva MC, Canevarolo RR, Alugubelli RR, DeAvila G, Tungesvik A*, et al.* A pharmacodynamic model of clinical synergy in multiple myeloma. *EBioMedicine* 2020 Apr; **54:** 102716.

62. Gatenbee CD, , Schenck RO, , Bravo RR, *, et al.* EvoFreq: visualization of the Evolutionary Frequencies of sequence and model data. Springer; 2019. p. 1-4.
