# Supplementary material for: The bone ecosystem facilitates multiple myeloma relapse and the evolution of heterogeneous drug resistant disease
Source: Nat Commun. 2024 Mar 19;15:2458. doi: 10.1038/s41467-024-46594-0 (PMC10951361; doi:10.1038/s41467-024-46594-0)
Supplement: Supplementary file 3 — Description of Additional Supplementary Files [file 41467_2024_46594_MOESM3_ESM.pdf]

Description of Additional Supplementary Files

Supplementary Movie Legends

Supplementary Movie 1. HCA model recapitulates normal bone remodeling. HCA simulation of normal bone remodeling, showing agent grid (top) and PDE grids with scaled concentration of RANKL (bottom left) and bone derived factors (BDF, bottom right) over one year. Each bone remodeling event is initiated through a small microfracture, which creates a signal to osteoblast lineage cells to express RANKL. Preosteoclasts migrate to the bone remodeling site and undergo fusion in response to RANKL to become a fully formed osteoclast. As the osteoclast resorbs the bone, BDF is released from the bone matrix which promotes the proliferation of preosteoblasts. During the reversal period, BDF continues to be released at a lower level, causing the preosteoblasts to migrate and attach to the eroded surface. Low BDF promotes the differentiation of preosteoblasts into bone-forming osteoblasts, which continue to build bone until the amount of bone that was resorbed is replaced. The bone formation phase is then followed by a period of quiescence until bone remodeling is re-initiated at the given location.

Youtube link: https://www.youtube.com/watch?v=UFpXzLNFMzo

Supplementary Movie 2. HCA model captures key steps of myeloma-bone vicious cycle. HCA simulation of the myeloma-bone vicious cycle, showing agent grid (top) and PDE grids with scaled concentration of RANKL (bottom left) and bone derived factors (BDF, bottom right) over one year. Each simulation is initialized with a single MM cell after the formation of the first osteoclast. MM increases the number of osteoclasts which drives bone resorption and the release of BDFs. BDFs and MSCs that are recruited in response to MM contribute to the growth and survival of MM cells (light green cells), causing MM cells to further expand in the bone marrow. At the same time, MM cells inhibit the differentiation of MSCs and preosteoblasts, preventing the formation of bone and further driving osteolytic bone disease.
Youtube link: https://www.youtube.com/watch?v=NFdpWp2l1wg

Supplementary Movie 3. EMDR increases minimal residual disease. HCA simulation of continuous treatment with bortezomib (BTZ), showing agent grid (top) and PDE grids with scaled concentration of RANKL (bottom left) and bone derived factors (BDF, bottom right). Treatment begins when multiple myeloma (MM) cells have reached 10% of the bone marrow (the starting point of the supplemental video) and continues until the MM burden doubles. After treatment begins, MM cells have a probability of resistance ($p\Omega$) during cell division that causes resistance to BTZ. When $p\Omega$ =10-4 , EMDR (middle) protected a portion of sensitive cells, enabling them to acquire resistance and drive tumor relapse. When EMDR is absent (right), the tumor went extinct.

Youtube link: https://www.youtube.com/watch?v=fnBuxXvWWXw

Supplementary Movie 4. Higher resistance probability increases tumor relapse rates in absence of EMDR HCA simulation of continuous treatment with bortezomib (BTZ), showing agent grid (top) and PDE grids with scaled concentration of RANKL (bottom left) and bone derived factors (BDF, bottom right). Treatment begins when multiple myeloma (MM) cells have reached 10% of the bone marrow (the starting point of the supplemental video) and continues until the MM burden doubles. After treatment begins, MM cells have a probability of resistance ($p\Omega$) during cell division that causes resistance to BTZ. When $p\Omega$ =10-3 , EMDR (middle) protected a portion of sensitive cells, enabling them to acquire

resistance and drive tumor relapse. When EMDR is absent (right), the high resistance probability prevents tumors from going extinct before the acquisition of intrinsic resistance and also drives tumor relapse.

Youtube link: https://www.youtube.com/watch?v=EJUOy0yepWw
