# Supplementary material for: The bone ecosystem facilitates multiple myeloma relapse and the evolution of heterogeneous drug resistant disease
Source: Nat Commun. 2024 Mar 19;15:2458. doi: 10.1038/s41467-024-46594-0 (PMC10951361; doi:10.1038/s41467-024-46594-0)
Supplement: Supplementary file 8 — Reporting Summary [file 41467_2024_46594_MOESM8_ESM.pdf]

nature portfolio

Corresponding author(s): Conor Lynch
David Basanta

Last updated by author(s): 2024/02/29

Reporting Summary

Nature Portfolio wishes to improve the reproducibility of the work that we publish. This form provides structure for consistency and transparency in reporting. For further information on Nature Portfolio policies, see our Editorial Policies and the Editorial Policy Checklist.

Statistics

For all statistical analyses, confirm that the following items are present in the figure legend, table legend, main text, or Methods section.

n/a	Confirmed	
☐	☒	The exact sample size (*n*) for each experimental group/condition, given as a discrete number and unit of measurement
☐	☒	A statement on whether measurements were taken from distinct samples or whether the same sample was measured repeatedly
☐	☒	The statistical test(s) used AND whether they are one- or two-sided<br>*Only common tests should be described solely by name; describe more complex techniques in the Methods section.*
☒	☐	A description of all covariates tested
☒	☐	A description of any assumptions or corrections, such as tests of normality and adjustment for multiple comparisons
☐	☒	A full description of the statistical parameters including central tendency (e.g. means) or other basic estimates (e.g. regression coefficient) AND variation (e.g. standard deviation) or associated estimates of uncertainty (e.g. confidence intervals)
☐	☒	For null hypothesis testing, the test statistic (e.g. *F*, *t*, *r*) with confidence intervals, effect sizes, degrees of freedom and *P* value noted<br>*Give P values as exact values whenever suitable.*
☒	☐	For Bayesian analysis, information on the choice of priors and Markov chain Monte Carlo settings
☒	☐	For hierarchical and complex designs, identification of the appropriate level for tests and full reporting of outcomes
☒	☐	Estimates of effect sizes (e.g. Cohen's *d*, Pearson's *r*), indicating how they were calculated

Our web collection on statistics for biologists contains articles on many of the points above.

Software and code

Policy information about availability of computer code

Data collection	In silico data collection: https://github.com/dbasanta/MM_ABM<br>Flow cytometry: BD FACSDIVA<br>uCT: SCANCO µCT 40<br>Microscopy: Zen 2 blue edition
Data analysis	In silico data analysis: https://github.com/dbasanta/MM_ABM<br>Statistics and Graphing software - Prism Version 10.1.0<br>Image Analysis: Zen 2 blue edition and Fiji version 1.0<br>Flow cytometry: FCS Express 7

For manuscripts utilizing custom algorithms or software that are central to the research but not yet described in published literature, software must be made available to editors and reviewers. We strongly encourage code deposition in a community repository (e.g. GitHub). See the Nature Portfolio guidelines for submitting code & software for further information.

Data

Policy information about availability of data

All manuscripts must include a data availability statement. This statement should provide the following information, where applicable:

- Accession codes, unique identifiers, or web links for publicly available datasets
- A description of any restrictions on data availability
- For clinical datasets or third party data, please ensure that the statement adheres to our policy

Biological data for all figures in this paper are provided in the accompanying Source data file. The raw images generated in this study are available freely by contacting either corresponding author Conor Lynch (conor.lynch@moffitt.org) or David Basanta (David@cancerevo.org). No publicly available or previously published datasets were used in this study. The in silico data generated in this study have been deposited in the Open Science Framework (OSF) database under identifier DOI 10.17605/OSF.IO/TNAX9115 [https://osf.io/tnax9/?view_only=7ff837c0a36e4b728791ec70f9935cd4]. The bone ecosystem facilitates multiple myeloma relapse and the evolution of heterogeneous drug resistant disease. Open Science Framework. DOI 10.17605/OSF.IO/TNAX9 (2023). Source data are provided with this paper.

Research involving human participants, their data, or biological material

Policy information about studies with human participants or human data. See also policy information about sex, gender (identity/presentation), and sexual orientation and race, ethnicity and racism.

Reporting on sex and gender	All patient data was provided to us through an honestbroker. Sex and gender were not considered as such we did not receive this individual information. 51.16% of samples were from males and 48.84% from females
Reporting on race, ethnicity, or other socially relevant groupings	All patient data was provided to us through an honestbroker. Race, ethinicity or other social groupings were not considered as such we did not receive this information.
Population characteristics	The myeloma specimens used represent a wide patient selection based on clinical demographics and are in accord with human subject protection, and scientific rigor in biomedical research.All patient data was provided to us through an honestbroker. Patients must have been diagnosed with relapsed/refractory multiple myeloma. Prior treatment was taken in to consideration; patients must have been treated with and failed a proteasome inhibitory containing regimen as their last treatment regimen. median age of patients was 65.1 years (range, 36.0-82.8 years)
Recruitment	Patients were recruited based on a diagnosis of relapsed/refractory multiple myeloma, having last failed a PI containing regimen and the capacity for informed consent.
Ethics oversight	Total Cancer Care® (protocols MCC14745 and MCC18608) at H. Lee Moffitt Cancer Center and Research Institute, as approved by the Institutional Review Board

Note that full information on the approval of the study protocol must also be provided in the manuscript.

Field-specific reporting

Please select the one below that is the best fit for your research. If you are not sure, read the appropriate sections before making your selection.

☒ Life sciences ☐ Behavioural & social sciences ☐ Ecological, evolutionary & environmental sciences

For a reference copy of the document with all sections, see nature.com/documents/nr-reporting-summary-flat.pdf

Life sciences study design

All studies must disclose on these points even when the disclosure is negative.

Sample size	With an alpha of 0.05, 4 mice per group achieves 80% power to detect a 75% reduction in trabecular bone volume (BV/TV) in myeloma bearing mice compared to tumor naïve mice. Similarly, 5 mice per group achieves 90% power to detect the expected bone loss. For these reasons, we chose to use a small, yet appropriate, number of mice for our experiments.<br>In vivo 2 sample sizes were based on prior studies and standards in the field (PMID: 27806331, PMID: 31442332)<br>In vitro studies were performed at least 3 times to esnure repoducibility. In silico simulations were performed at least 25 times.
Data exclusions	Two mice from in vivo study 1 in the U266 bearing group day 40 were not analysed/excluded from uCT/IF and histomorphometry owing to breaking of the tibia on excision.<br>One experiment from 6L/7D was given DAPI to look for dead/live cells. This data was not included but the live cell counts after this were not included since no other experiments had been given DAPI.
Replication	All in vitro studies were independently repeated on at least 3 separate occasions. All repeats were successful.<br>Animal study 1 was performed once since there were striking difference between normal bone and tumor bearing bones and it was not necessary to waste more mice. The second animal experiment was performed only once as part of the review process.

Randomization	For experiment 1, all animals were allocated in to groups prior to injection of tumor cells or PBS based on age and sex matching to ensure even distribution of these variables.<br>For experiment 2, all mice that were injected with tumor cells were then allocated to 4 groups such that the median luminesence signal was similar between groups.<br>No randomization was performed for in vitro experiments nor in silico experiments as it is impractical/prone to errors or not possible. Bespalov, A., Wicke, K., Castagné, V. (2019). Blinding and Randomization. In: Bespalov, A., Michel, M., Steckler, T. (eds) Good Research Practice in Non-Clinical Pharmacology and Biomedicine. Handbook of Experimental Pharmacology, vol 257. Springer, Cham. https://doi.org/10.1007/164_2019_279
Blinding	Investigators were blinded to mice groupings prior to uCT and IF or hisitochemical analyses and unblinded following analysis. Investigators were not blinded to mice groupings at the time of study to facilitate correct treatments.<br>No blinding was performed for in vitro or in silico experiments. The authors were not blinded to group allocation, data collection or data analysis because the investigators were responsible for performing the experiment, collecting and labelling the samples, and analysis of data in invitro and in silico experiments.

Reporting for specific materials, systems and methods

We require information from authors about some types of materials, experimental systems and methods used in many studies. Here, indicate whether each material, system or method listed is relevant to your study. If you are not sure if a list item applies to your research, read the appropriate section before selecting a response.

Materials & experimental systems

n/a	Involved in the study
☐	☒ Antibodies
☐	☒ Eukaryotic cell lines
☒	☐ Palaeontology and archaeology
☐	☒ Animals and other organisms
☒	☐ Clinical data
☒	☐ Dual use research of concern
☒	☐ Plants

Methods

n/a	Involved in the study
☒	☐ ChIP-seq
☐	☒ Flow cytometry
☒	☐ MRI-based neuroimaging

Antibodies

Antibodies used	Anti-pHH3 (Cat# 06-570) Millipore, Lot # 2972863 (1 in 200)<br>Anti-Osterix (Cat# ab209484) Abcam, Lot# GR3263824-6 (1in 500)<br>Anti-aSMA: PA5-16697, Invitrogen (1 in 200)<br>AlexaFluor 647 Goat anti-rabbit IgG (H+L) Invitrogen (#A21244) lot# 2277746 (1 in 1000)
Validation	anti-pHH3 (Cat# 06-570) Millipore was validated for Immunocytochemistry Analysis: 1:500 dilution from a representative lot detected Histone H3 in HeLa and A431 cells and by WB and IP.<br><br>Anti-Osterix (Cat# ab209484) was validated by Abcam: Immunohistochemical analysis of paraffin-embedded rat E14.5 rib tissue labeling Sp7 / Osterix with ab209484 at 1/1000 dilution, followed by Goat Anti-Rabbit IgG H&L (HRP) Ready to use. Nuclear staining on osteoblasts and chondrocytes of rat E14.5 rib (PMID: 17579353; PMID: 25977369) is observed.<br><br>anti-aSMA: PA5-16697, Invitrogen was validated by Invitrogen: Immunofluorescent analysis of Actin Smooth Muscle was performed using 70% confluent log phase C2C12 cells. The cells were fixed with 4% paraformaldehyde for 10 minutes, permeabilized with 0.1% Triton™ X-100 for 10 minutes, and blocked with 1% BSA for 1 hour at room temperature. The cells were labeled with Actin Smooth Muscle Rabbit Polyclonal Antibody (Product # PA5-16697) at 2 µg/mL in 0.1% BSA and incubated for 3 hours at room temperature<br><br>Rabbit IgG (H+L) Cross-Adsorbed Secondary Antibody (A-21244) in ICC/IF<br>Immunofluorescence analysis of Goat anti-Rabbit IgG (H+L) Cross-Adsorbed Secondary Antibody, Alexa Fluor® 647 conjugate was performed using HeLa cells stained with alpha Tubulin Rabbit Polyclonal Antibody (Product # PA5-16891). The cells were fixed with 4% paraformaldehyde for 10 minutes, permeabilized with 0.1% Triton™ X-100 for 10 minutes, blocked with 1% BSA for 1 hour and labeled with 2 µg/mL primary antibody for 3 hours at room temperature. Goat anti-Rabbit IgG (H+L) Cross-Adsorbed Secondary Antibody, Alexa Fluor® 647 conjugate (Product # A-21244) was used at a concentration of 4 µg/mL in phosphate buffered saline containing 0.2% BSA for 45 minutes at room temperature, for detection of alpha Tubulin in the cytoplasm (Panel a: red). Nuclei (Panel b: blue) were stained with DAPI in SlowFade® Gold Antifade Mountant (Product # S36938). F-actin was stained with Alexa Fluor® 488 Phalloidin (Product # A12379), 1:300) (Panel c: green). Panel d represents the composite image. No nonspecific staining was observed with the secondary antibody alone (panel f), or with an isotype control (panel e). The images were captured at 60X magnification.

Eukaryotic cell lines

Policy information about cell lines and Sex and Gender in Research

Cell line source(s)	Human U266 (RRID:CVCL_0566) and U266-PSR (a bortezomib resistant derivative) were kind gifts from Dr. Steven Grant.

Cell line source(s)	MC3T3-E1 (CRL-2594) were purchased from ATCC.<br>Human MSCs (PT-2501 #44630 male) were purchased from Lonza.<br>Murine MSCs and bone marrow macrophages were isolated from 6-week old male and female RAG2-/- mice.
Authentication	Myeloma cell lines were authenticated by short tandem repeat (STR) verified at the Moffitt Clinical Translational Research Core. Human and murine MSCs were not authenticated by STR but have shown the ability to form in to osteoblasts and adipocytes
Mycoplasma contamination	All cell lines were periodically mycoplasma tested (CUL001B, R&D Systems) and were negative.
Commonly misidentified lines (See ICLAC register)	No commonly misidentified cell lines were used.

Animals and other research organisms

Policy information about studies involving animals; ARRIVE guidelines recommended for reporting animal research, and Sex and Gender in Research

Laboratory animals	8 week and 14-16-week old male and female NSG mice were used for in vivo studies 2 and 1 respectively.<br>6 week old C57BL6 RAG2-/- male and female mice were used for the isolation of bone marrow cells.<br>Animals were housed in a 12-hour light/dark, ambient temperature and humidity housing facility.
Wild animals	Study did not involve wild animals
Reporting on sex	Myeloma affects both sexes however the incidence is higher in males than females (57% vs 43%) PMID: 34059488. As such both sexes were used. Both male and female mice developed myeloma and subsequent bone disease. Our findings are relevant to both sexes and as such we have not shown disaggregated sex data.
Field-collected samples	Study did not involve field collected samples
Ethics oversight	All animal experiments were performed with University of South Florida IACUC approval #7356R, #10955R

Note that full information on the approval of the study protocol must also be provided in the manuscript.

Plants

Seed stocks	n/a
Novel plant genotypes	n/a
Authentication	n/a

Flow Cytometry

Plots

Confirm that:

☒ The axis labels state the marker and fluorochrome used (e.g. CD4-FITC).

☒ The axis scales are clearly visible. Include numbers along axes only for bottom left plot of group (a 'group' is an analysis of identical markers).

☒ All plots are contour plots with outliers or pseudocolor plots.

☒ A numerical value for number of cells or percentage (with statistics) is provided.

Methodology

Sample preparation	Ex vivo analysis: Left tibiae were used to assess tumor burden by GFP expression. Tibial ends were excised, whole bone marrow was isolated by centrifugation at 10,000g for 10 seconds.<br>Red blood cells were lysed by RBC lysis buffer (R7757, Sigma-Aldrich) as per manufacturers guidelines. Bone marrow cells were subject to viability staining with Zombie Near-Infrared (NIR; 1:500; 423105, BioLegend). Appropriate compensation and fluorescence-minus-one (FMO) controls were generated in parallel either with aliquots of bone marrow cells or U266GFP+/-<br>To address the proliferative advantages provided by cells of the BME to MM cells, U266 MM cells were stained with

CM-DIL (Invitrogen; V22888) according to the manufacturer's instruction and incubated with 50% (v/v) control, MSC, pOBs (Day 7 or 14) or OB (day 21 or 28) conditioned media for 7 days, with media changes every 3 days. An aliquot of untreated CM-DIL+ cells was stained with Zombie NIR as above and used for baseline time point. At the end of the experiment, MM cells were stained with Zombie NIR for live/dead discrimination. The MFI for the CM-DiI channel was calculated at baseline and after 72 hours on live single cell cells using appropriate FMO controls.

Instrument

BD Biosciences LSRII flow cytometer

Software

Acquisition - BD FACSDIVA v. 6.1.3
Analysis - FCS Express v7

Cell population abundance

No sorting was performed.

Gating strategy

Cell Morphology (FSC-A x SSC-A) -> FSC singlets (FSC-W x FSC-H) -> SSC Singlets (SSC-W x SSC-H) -> Live cells [Zombie NIR negative cells] (FSC-A x ZOMBIE NIR) -> GFP+ MM cells (GFP x FSC-A)
Cell Morphology (FSC-A x SSC-A) -> FSC singlets (FSC-A x FSC-H) -> SSC Singlets (SSC-A x SSC-H) -> Live cells [DAPI negative cells] (FSC-A x DAPI) -> GFP+ OR RFP+ MM cells (GFP x RFP)
FMOs were used to select gating strategy.

☒ Tick this box to confirm that a figure exemplifying the gating strategy is provided in the Supplementary Information.
